# The LncRNA STEAP3-AS1 promotes liver metastasis in colorectal cancer by regulating histone lactylation through chromatin remodelling

**DOI:** 10.1186/s13046-025-03461-0

**Published:** 2025-07-15

**Authors:** Jinjuan Lv, Xiaoqi Yu, Xiaoqian Liu, Qianshi Zhang, Mengyan Zhang, Jianfeng Gao, Zhiwei Sun, Feifan Zhang, Yunfei Zuo, Shuangyi Ren

**Affiliations:** 1 Department of General Surgery, The Second Affiliated Hospital of Dalian Medical University, Dalian, 116023 China; 2 Department of Clinical Biochemistry, College of Laboratory Diagnostic Medicine, Dalian Medical University, Dalian, 116044 China; 3 Department of Clinical Laboratory Medicine, Xijing Hospital, Fourth Military Medical University, Xi’an, China

**Keywords:** Metastasis, LncRNA STEAP3-AS1, Chromatin remodelling, H3K18la

## Abstract

**Introduction:**

Liver metastasis is a common cause of death in patients with colorectal cancer (CRC); however, its molecular mechanism remains unclear. Here, we aimed to reveal the role of the lncRNA STEAP3-AS1 in regulating chromatin remodelling and histone lactylation and explore the mechanism by which the lncRNA STEAP3-AS1 promotes CRC liver metastasis. This study provide new research ideas and a theoretical basis for the clinical treatment of cancer.

**Methods:**

In this study, we used CRC organoid and nude mouse liver metastasis models to analyse the effect of the lncRNA STEAP3-AS1 on CRC liver metastasis. ATAC-seq, RNA-seq, DRIP-seq and Western blotting were used to screen for the lncRNA STEAP3-AS1 downstream prometastatic molecule MMP9 and the chromatin remodelling factor BRG1. The protein interactions between BRG1, p300, and HDAC3 were evaluated by Co-IP. The the binding of BRG1, ERG, P300, and H3K18la to the MMP9 gene promoter was detected using ChIP-qPCR.

**Results:**

The lncRNA STEAP3-AS1 interacts with its parental gene, STEAP3, to form an R-loop on the key chromatin remodelling factor BRG1, regulating the expression of BRG1. Further evidence suggests that BRG1 forms a protein complex with the histone lactylation eraser HDAC3 and the writer P300 to regulate the expression of H3K18la. Moreover, the ATAC-seq analysis revealed that the lncRNA STEAP3-AS1 promotes the chromatin accessibility of MMP9, and a motif and database analysis identified the tumour metastasis factor ERG as an MMP9 transcription factor. The lncRNA STEAP3-AS1 mediates regulation of H3K18la activation of MMP9 by the BRG1/ERG/P300 complex.

**Conclusions:**

In summary, these findings revealed that the lncRNA STEAP3-AS1 interacts with its parental gene STEAP3 to regulate H3K18la through BRG1, resulting in changes in chromatin accessibility, thereby driving ERG enrichment an the MMP9 promoter to activate MMP9 transcription and promote CRC liver metastasis. Our findings reveal a novel mechanism by which the lncRNA STEAP3-AS1 promotes CRC metastasis from an epigenetic perspective.

**Supplementary Information:**

The online version contains supplementary material available at 10.1186/s13046-025-03461-0.

## Introduction

Colorectal cancer (CRC) is the third most common type of malignant tumor and the second leading cause of cancer-related death [1]. The liver is the most common anatomical site of haematogenous metastasis of CRC. Approximately 21–26% of patients present with synchronous metastatic diseases, 14.5–17.5% of whom develop liver metastases [2]. Metastatic CRCs are associated with poor survival rates, and CRC-derived liver metastasis is a significant clinical challenge [3].

At present, research on CRC liver metastasis has focused mainly on heterogeneous cancer stem-like cells [4], the crosstalk between cancer cells and tumour-associated macrophages [5], and the epithelial‒mesenchymal transition (EMT) [6]. However, a study suggested that the complex mechanism of colorectal cancer metastasis is closely related to lncRNA-mediated epigenetics. Recent studies have shown that the lncRNA NR_109 mediates chromatin remodelling in M2-like macrophages, competing with JVT-1 to bind to the C-terminal domain of upstream element binding protein 1 (FUBP1), preventing the ubiquitin-mediated degradation of FUBP1, activating c-Myc transcription, promoting polarization of M2-like macrophages, and supporting tumour cell proliferation and metastasis in vitro and in vivo [7]. The study also revealed that the lncRNA TGFB2-AS1 interacts with brahma-related gene-1 (BRG1) and blocks the ability of the chromatin remodelling complex to approach its target promoters both in cis and in trans, thus inhibiting the expression of the target genes TGFB2 and SOX2, and eventually leading to the inhibition of breast cancer progression and lung metastasis [8]. However, whether lncRNAs mediate chromatin remodelling and play a role in the metastasis of colorectal cancer needs further study.

The SWItch sucrose nonfermentable (SWI/SNF) complex is an ATP-dependent chromatin remodelling complex that plays important roles in gene transcription and tumour metastasis [9]. BRG1 is an ATPase and core subunit of the human SWI/SNF chromatin remodelling complex that is associated mainly with gene transcription to regulate the biological function of cells. Lanbo Xiao et al. used the degradation agent AU-15,330 to target and reduce the expression of BRG1 in prostate cancer cell lines, leading to the detachment of the transcription factor ERG from chromatin and the inactivation of downstream oncogenes, thereby inhibiting prostate cancer growth [10]. The chromatin remodelling factor BRG1 forms a BRG1-EP300 complex with the histone acetyltransferase EP300, driving the expression of proliferation-related genes and affecting tumour progression [11]. Research has shown that knocking out BRG1 increases R-loop formation, leading to transcriptional replication conflicts caused by R-loop-dependent DNA breaks. This study reveals a new mechanism by which chromatin remodelling protects genome integrity [12]. However, the detailed molecular mechanism by which BRG1 mediates chromatin remodelling to promote tumour occurrence and metastasis still needs to be further elucidated.

Dysregulation of histone modifications can shift the balance of transcriptional activation and repression, resulting in the occurrence and progression of tumours [13]. Zhang et al. identified the lactate-derived lactylation of histones as a newly discovered epigenetic modification that activates gene transcription [14]. Research has shown that histone lactylation is significantly higher in ocular melanoma tissue than in normal tissue. H3K18la is enriched at the promoter region of the YTH N6-methyladenosine RNA-binding protein 2 (YTHDF2) gene, activating the expression of YTHDF2 and promoting tumorigenesis [15]. Recent studies have shown that zinc finger E-box binding homeobox 1 (Zeb1) upregulates the expression of key glycolytic enzymes (HK2, PFKP, and LDHA), leading to lactate accumulation and increased histone lactylation, thereby increasing chromatin accessibility and cell lineage plasticity and promoting the development of prostate neuroendocrine carcinoma [16]. Therefore, histone lactylation is closely related to disease onset and development, and the exploration of the role of histone lactylation in the pathogenesis of diseases, especially tumorigenesis, has attracted increasing attention.

A key stage in promoting tumour metastasis is the degradation of the extracellular matrix (ECM) by matrix metalloproteinases (MMPs). Matrix metalloproteinase-9 (MMP9) is one of the most complex matrix metalloproteinases. MMP9 plays an important role in pathophysiological functions [17, 18]. The overexpression and dysregulation of MMP9 are associated with various diseases. SE translocation (SET) reportedly interacts with zinc finger and BTB domain containing 11 (ZBTB11) to promote the complex-mediated transcriptional activation of MMP9, which promotes lung cancer distal metastasis [19]. Studies have also shown that GATA binding protein 4 (GATA4) recruits histone deacetylase, resulting in reduced acetylation of the transcription factor p65, thereby inhibiting p65 binding to the MMP9 promoter, inhibiting the transcriptional activity of MMP9, and preventing breast cancer metastasis [20]. In addition, TGFβ/TNFα costimulates the MMP9 promoter H3K36me2 through chromatin remodelling to activate MMP9 transcription and promote the invasion and metastasis of breast cancer [21]. Therefore, regulating and inhibiting MMP9 is an important strategy for treating various diseases, especially cancer.

In this study, we focused on the function of lncRNA STEAP3-AS1-mediated chromatin remodelling in colorectal cancer and explored the mechanism of action. Liver metastasis models and organoid models of colorectal cancer were established in vitro and in vivo. Notably, the lncRNA STEAP3-AS1 promotes the growth of organoids and liver metastasis of CRC. Mechanistic research has indicated that the lncRNA STEAP3-AS1 mediates the BRG1/H3K18la/ERG complex, promoting MMP9 transcription and promoting CRC liver metastasis; thus, the lncRNA STEAP3-AS1 can be considered a novel metastasis-promoting molecule in CRC.

## Materials and methods

### Patients and tissue samples

We used 15 human colorectal cancer tissues, corresponding adjacent normal tissues, and matched liver metastatic tissues obtained through surgical resection from patients without preoperative treatment at the Second Hospital of Dalian Medical University (Dalian, China) between 2021 and 2023. With the approval and support of the Ethics Committee of the Second Hospital of Dalian Medical University, the diagnosis of colorectal cancer was confirmed by a pathologic examination. Informed consent was obtained from all the patients. The samples used for FISH and IHC staining were FFPE (formalin-fixed and paraffin-embedded) samples.

### Cell culture

Human colorectal cancer (HCT-116 and LoVo) and normal human intestinal epithelial (HCoEpiC) cells were purchased from the Institute of Biochemistry and Cell Biology of the Chinese Academy of Sciences (Shanghai, China). The HCT-116 cells were cultured in RPMI-1640 (Gibco) medium, and the LoVo and HCoEpiC cells were cultured in DMEM (Gibco). The cells were cultured in medium supplemented with 10% certified heat-inactivated foetal bovine serum (Gibco), penicillin (100 U/mL), and streptomycin (100 mg/mL) at 37 °C in a humidified 5% CO_2_ atmosphere.

### In vivo metastasis assay

All the experimental procedures were conducted in accordance with the institutional guidelines for the care and use of laboratory animals, and the protocols were approved by the Institutional Animal Care and Use Committee of Dalian Medical University Laboratory Animal Center. BALB/c-nu mice that were 4–6 weeks of age were purchased from Vital River (Beijing, China). At the age of 4 weeks, the mice (*n* = 5) were divided into three groups: (1) control, (2) lncRNA STEAP3-AS1 shRNA1, and (3) lncRNA STEAP3-AS1 shRNA2. Briefly, 2 × 10^6^ CRC cells (HCT-116 or LoVo) transduced with lentiviral vectors were injected into the liver or spleen capsules (Glisson’s capsules) of recipient mice. The liver or spleen was returned to the abdominal cavity, and the incision was closed. The mice were sacrificed at 3–7 weeks postinjection and examined microscopically by H&E staining for the development of liver metastatic foci.

### ATAC-seq library preparation and differential accessibility

Using LoVo cells (5 × 10^4^ cells), libraries were purified using AMPure beads (Beckman, A63881) and sequenced using the Illumina NovaSeq platform with paired ends (PE150) at Annoroad Gene Technology. Bowtie2 software was used to compare the sequencing data with the reference genome, filter the reads that were compared to the organelle genome and the reads that were repeatedly compared, and retain high-quality perfectly matched double-ended reads for analysis. ATAC-seq peaks were identified using MACS257. Coverage tracks were generated from aligned reads using DeepTools with RPKM normalization (version 3.5.0) 58. Differential accessibility was assessed using DESeq2. Unless stated otherwise, regions were considered differentially accessible if the absolute value of the log2 fold change was > 0.5 at an FDR < 0.1. The tracks were displayed using Integrative Genomics Viewer46.

### DNA‒RNA immunoprecipitation (DRIP)

A total of 5 × 10^7^ HCT-116 cells were collected using trypsin, and the cell suspension was treated with protease K (20 µg/mL) and 0.5% SDS at 37 °C overnight. Genomic DNA was extracted by mixing equal volumes of phenol/chloroform/isoamyl alcohol solutions. Total DNA was segmented using restriction enzymes (HindIII, EcoRI, XbaI, and BamHI). The DNA fragments were purified using phenol‒chloroform–isopropyl alcohol, and their concentration and quality were recorded. As a negative control, the sample was combined with 3 µL of RNH. A sample of 4 µg of digested RNA (with or without RNH treatment) was incubated with 2.5 µL of an S9.6 antibody in 500 µL of binding buffer at 4 °C overnight. Oligonucleotide‒protein complexes were immunoprecipitated with Protein A magnetic beads (Bio-Rad Laboratories) at 4 °C for 2 h, washed three times with binding buffer, and eluted with elution buffer (50 mM Tris, pH 8.0, 10 mM EDTA, and 0.5% SDS) and proteinase K (1.4 mg/mL) at 55 °C for 45 min. The sample was sequentially mixed with ammonium acetate (0.75 M) and glycogen (20 µg) and extracted with 2.5 volumes of cold ethanol. The air-dried pellets were dissolved in water. High-throughput sequencing of the obtained samples was performed. The results were analysed using QuantStudio™ real-time PCR software (Applied Biosystems, Foster City, California, USA). The sequences of primers used in this study are listed in Supplementary Table S2. The qPCR conditions were as follows: 95 °C for 10 min and 40 cycles of 95 °C for 15 s and 60 °C for 1 min.

### Patient-derived organoid (PDO) culture

Fresh tumour tissues collected from CRC patients were washed three times with ice-cold PBS containing penicillin‒streptomycin, minced into small pieces (3–5 mm) quickly, digested with organoid digestion solutions I and II, and digested at 37 °C for 2 h and 10 min, respectively. When the cells were observed to be in clumps under a microscope, the digestion was complete. The cells were mixed with Matrigel (8 mg/mL; Corning) and seeded in a 24-well plate. Matrigel polymerization mixture (30 min at 37 °C) was added to 500 µL of Colon Cancer Organ Culture Medium (Cat.# M102, Accurate International). The organoids were passaged for approximately two weeks, and passage number 2 was used in further studies. Matrigel-embedded organoids were incubated with 100 µL of plasma and 50 µL of thrombin (20 IU/ml, Siemens), fixed overnight with 4% formaldehyde and then sectioned for H&E staining.

### Statistical analysis

Each experiment was performed at least three times. The data are presented as the means ± SDs. Differences between two groups were assessed using two-tailed Student’s t tests. Multiple group comparisons were performed using one-way analysis of variance (ANOVA). The paired samples were analysed using paired t tests. A *p* value of less than 0.05 was considered statistically significant (*P < 0.05, **P < 0.01, and ***P < 0.001). Details of the specific statistical tests can be found in each figure legend. All the statistical analyses were performed using GraphPad Prism software (version 8.0).

## Results

### Silencing of the highly expressed LncRNA STEAP3-AS1 in CRC specifically impairs the organoid growth and liver metastasis of CRC

**Fig. 1:**
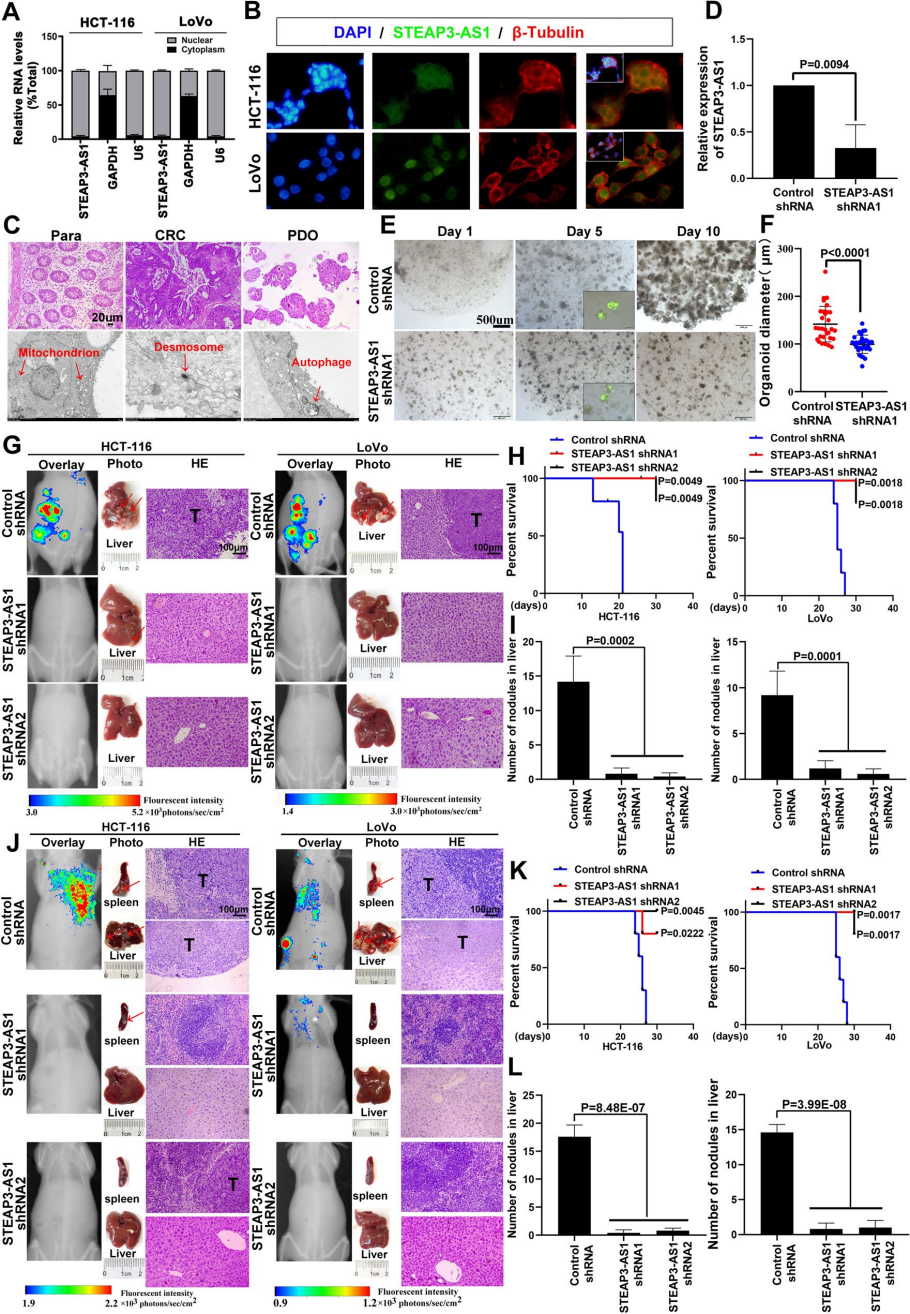
LncRNA STEAP3-AS1 silencing inhibits the growth of organoids and the liver metastasis of CRC. (**A** and **B**) Nucleoplasmic isolation (**A**) and FISH (**B**) assays verified that the lncRNA STEAP3-AS1 was localized in the nucleus. (**C**) CRC organoids cultured in vitro were evaluated using H&E staining and transmission electron microscopy. Scale bars for images of H&E staining, 100 μm. (**D**) RT‒qPCR analysis of the efficiency of lncRNA STEAP3-AS1 knockdown in organoids. (**E**) Brightfield imaging of organoid growth after control or lncRNA STEAP3-AS1 shRNA1 silencing. Scale bars, 500 μm. (**F**) ImageJ was used to analyse the diameter of organoid growth. (**G**) Fluorescence analysis of the kinetics of metastases of the indicated cells in the liver injection model (left panel). The livers were examined at the gross anatomical level for tumours and metastases (middle panel). Representative image of H&E staining showing metastatic nodules (right panel). T, tumour. (**H**) Kaplan–Meier survival curves of mice with lncRNA STEAP3-AS1 knockdown cells in the liver injection model. (**I**) The number of metastatic nodules in the liver of the liver injection model mice. (**J**) Fluorescence analysis of the kinetics of metastases of the indicated cells in the splenic injection model mice (left panel). The livers were examined at the gross anatomical level for tumours and metastases (middle panel). Representative image of H&E staining showing metastatic nodules (right panel). T, tumour. (**K**) Kaplan–Meier survival curves for mice implanted with cells in which lncRNA STEAP3-AS1 was knocked down in the splenic injection model. (**L**) The number of metastatic nodules in the livers of the splenic injection model mice. Representative data from three (E) or five (G and J) independent experiments are shown

In previous research, we found that dendritic cell-specific intercellular adhesion molecule-grabbing nonintegrin-related protein (DC-SIGNR), a type of C-type lectin, increased the expression of the metallothionein MT1M during colon cancer progression, and we suspected that metallothioneins may be associated with colon cancer [22]. Therefore, the LncPath human cancer chip was used to screen for lncRNAs related to MT1M, and the lncRNA STEAP3-AS1 was observed to be significantly overexpressed in colorectal cancer, promoting the proliferation and migration of colorectal cancer cells in vitro and the growth of colorectal cancer in vivo [23]. These findings demonstrate the role of the lncRNA STEAP3-AS in promoting cancer. We further analysed the differential expression of the lncRNA STEAP3-AS1 in colon cancer tissues using the GEO database (GSE109454). The results showed that the lncRNA STEAP3-AS1 was significantly overexpressed in CRC samples (Figure S1A). Moreover, we detected the subcellular localization of the lncRNA STEP3-AS1. The results revealed that the lncRNA STEAP3-AS1 was localized mainly in the nucleus of cells, indicating that the lncRNA STEAP3-AS1 plays a biological role in the nucleus (Fig. 1A and B, Figure S1B). Transwell migration assays confirmed that lncRNA STEAP3-AS1 overexpression markedly increased the migratory potential of CRC cells (Figure S1C). Colony formation, Cell Counting Kit-8 (CCK‐8), and wound healing assays revealed a significant increase in the growth of CRC cells after overexpression of the lncRNA STEAP3-AS1 compared with the negative control (Figure S1D‒G). Together, these results suggest that the lncRNA STEAP3-AS1 promotes the migration and proliferation of colon cancer cells. We next established a model of CRC patient-derived organoids (PDOs) (Fig. 1C, Figure S1H). Compared with the CRC tissue, the organoids were highly proliferative spheroids with robust Ki-67 and CK20 staining (Figure S1I and J). Two short hairpin RNAs (shRNAs) targeting the lncRNA STEAP3-AS1 were designed to silence the expression of the lncRNA STEAP3-AS1 via lentiviral transduction and the effect of this lncRNA on the proliferation of organoids was investigated. As shown in Fig. 1D, we successfully constructed organoids in which the lncRNA STEAP3-AS1 was knocked down. The results revealed that the control group exhibited high proliferation in the organoids, but lncRNA STEAP3-AS1 knockdown inhibited organoid growth and weakened Ki-67 staining (Fig. 1E and F, Figure S1K and L). In addition, we constructed a lncRNA STEAP3-AS1 KO cell line (Figure S1M), and both colony formation and CCK-8 assays revealed that knocking out lncRNA STEAP3-AS1 significantly inhibited the proliferation of colorectal cancer cells, which was consistent with the results of the organoid experiments (Figure S1N and O).

Stable CRC cells expressing the negative control shRNA or an shRNA designed to knock down the lncRNA STEAP3-AS1 were injected into the livers of nude mice to determine whether the lncRNA STEAP3-AS1 mediates CRC metastasis to specific organs. All the mice injected with HCT-116 or LoVo cells expressing the control shRNA exhibited significant fluorescence in the liver and metastatic tumours formed. In contrast, the shRNA-mediated knockdown of the lncRNA STEAP3-AS1 in HCT-116 and LoVo cells rarely resulted in liver metastasis in tumour-bearing mice (Fig. 1G). The survival analysis revealed that the survival rate of mice injected with lncRNA STEAP3-AS1-knockdown cells was significantly improved (Fig. 1H). In addition, compared with those in the control group, the number of metastatic nodules in the liver was significantly reduced in the lncRNA STEAP3-AS1-knockdown shRNA group (Fig. 1I). We established a metastasis model by injecting cells at the distal tip of the spleen to further clarify the effect of the lncRNA STEAP3-AS1 on the liver metastasis of CRC. Interestingly, the results were consistent with those of the liver injection experiments, and lncRNA STEAP3-AS1 knockdown significantly inhibited the liver metastasis of colorectal cancer (Fig. 1J) and thereby increased the survival time of nude mice, indicating that inhibiting the lncRNA STEAP3-AS1 significantly inhibited the targeted liver metastasis of CRC (Fig. 1K and L). IHC staining showed that the expression of the marker molecules Ki-67 and CK20 for CRC metastasis was strongly positive in the control group, whereas no significant change was observed in the knockdown group (Figure S1P). Together, these results suggest that lncRNA STEAP3-AS1 knockdown can significantly impair the proliferation, migration, organoid growth, and liver metastatic ability of CRC cells both in vitro and in vivo.

### LncRNA STEAP3-AS1-mediated chromatin remodelling promotes the liver metastasis of CRC

**Fig. 2:**
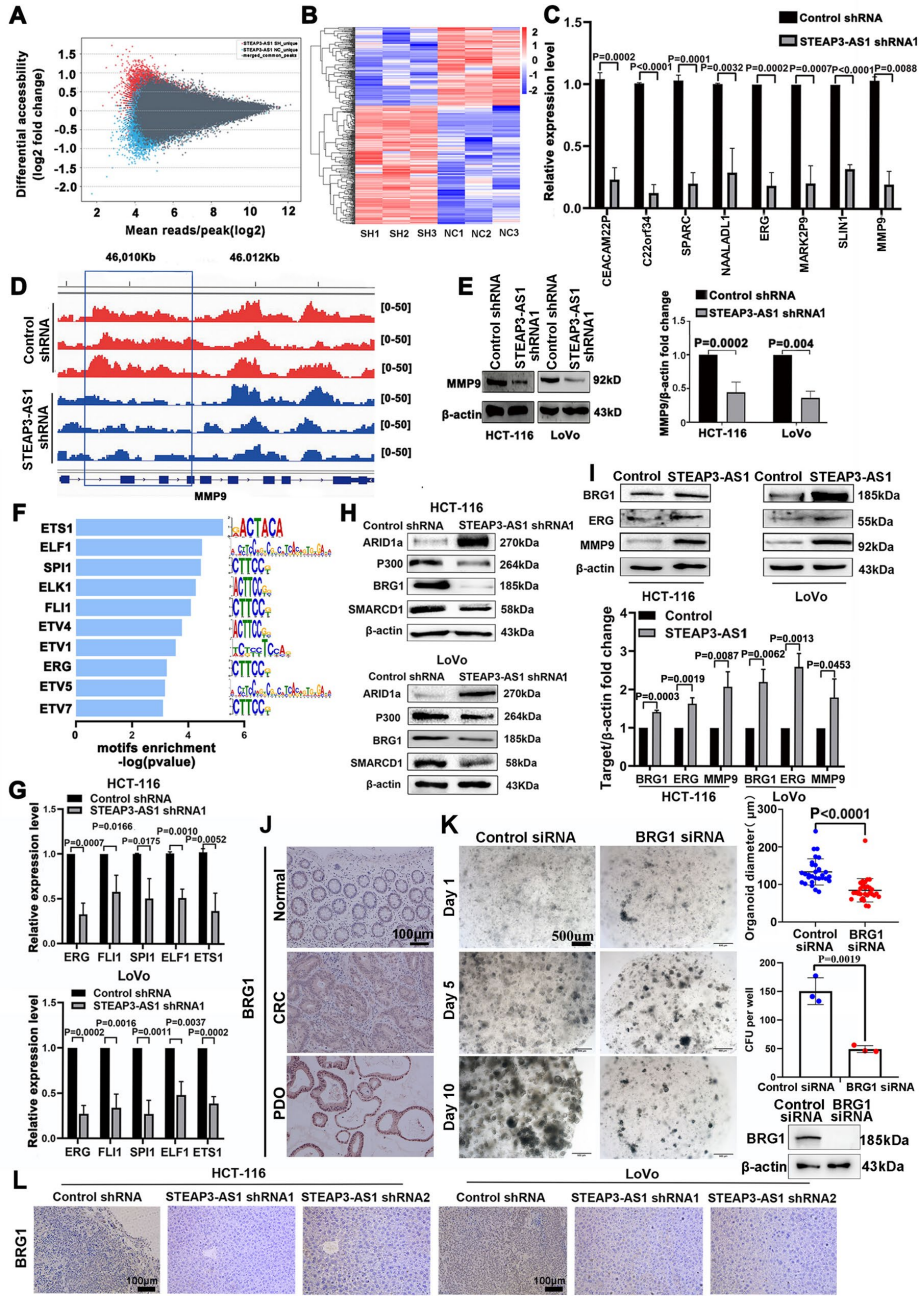
The lncRNA STEAP3-AS1 regulates specific genes associated with the induction of MMP9 gene expression. (**A**) Differences in gene accessibility. (**B**) Differential gene expression heatmaps; blue indicates lower expression levels, and red indicates higher expression levels. (**C**) The RT‒qPCR analysis revealed MMP9 as a downstream target of the lncRNA STEAP3-AS1 (fold change ≥ 2). (**D**) IGV tracks for MMP9 from the ATAC-seq analysis. (**E**) Western blotting was performed to verify the expression of MMP9 after the lncRNA STEAP3-AS1 was knocked down. (**F**) ATAC-seq motif analysis of the enrichment of ETS family transcription factors in the open chromatin region of the genome. (**G**) RT‒qPCR analysis of the regulatory effect of the lncRNA STEAP3-AS1 on ETS family transcription factors. (**H**) Western blot analysis of the protein levels of key molecules involved in chromatin remodelling. (**I**) Western blotting was performed to detect the expression of the BRG1, ERG and MMP9 proteins after lncRNA STEAP3-AS1 overexpression. (**J**) IHC analysis of BRG1 expression in normal and CRC tissues and organoids. Scale bars, 100 μm. (**K**) Bright-field images of organoid growth after control or BRG1 siRNA transfection. Scale bars, 500 μm. (**L**) IHC analysis of the expression of BRG1 in the liver metastatic tissues from the liver injection model mice. Scale bars, 100 μm

Previous studies have shown that the lncRNA STEAP3-AS1 affects the expression of CDKN1C, CDK2, and CDK4 and histone H3 acetylation, which are involved in regulating cell cycle progression in colon cancer [23]. We speculate that the lncRNA STEAP3-AS1 mediates chromatin remodelling in CRC cells. For the ATAC-seq project, based on peak detection and a comparison of genomic information, the MANORM-PE model was used to identify the difference peaks between the comparison groups, and the difference peaks were ultimately obtained (Fig. 2A, Figure S2A). The RNA-seq analysis identified 455 differentially expressed genes, of which 181 were downregulated (adj. *p* ≤ 0.05,| log 2 (fold change)|≥1) (Fig. 2B). GO and KEGG analyses revealed that these lncRNA STEAP3-AS1-specific genes were enriched in tumour-related pathways (Figure S2B and C), suggesting that the lncRNA STEAP3-AS1 has a regulatory effect on tumorigenesis. Through a combined analysis of RNA-seq and ATAC-seq data, downregulated mRNA expression of 17 genes was identified (Figure S2D). We performed RT‒qPCR to validate genes with a fold change in expression ≥ 2 among the eight downregulated genes (Fig. 2C). We found that MMP9 was significantly downregulated and showed the most significant change in chromatin accessibility after knocking down the lncRNA STEAP3-AS1 (Fig. 2D). MMP9 is involved in the development of the tumour microenvironment and is closely related to tumour metastasis [24]. The findings of the aforementioned animal experiments suggested that the lncRNA STEAP3-AS1 promoted CRC liver metastasis and that the change in MMP9 gene accessibility was the most significant; thus, we focused on the MMP9 molecule. Further investigation revealed that the knockdown of the lncRNA STEAP3-AS1 significantly reduced the protein expression level of MMP9 (Fig. 2E, Figure S2E). We employed transcription factor footprinting to investigate whether the lncRNA STEAP3-AS1 could lead to chromatin accessibility of the MMP9 gene [25]. Motif sequences in different regions of gene accessibility and the open region were screened, and the top 10 motifs bound to DNA sequences were listed. The results revealed that EST family enrichment was most significant (Fig. 2F). Therefore, we speculated that the ETS family of transcription factors may be a key driving factor through which the lncRNA STEAP3-AS1 regulates MMP9. We combined the results of the analyses of the hTFtarget, TFBD, and JASPAR databases with the ATAC-seq motif analysis results to screen for five possible ETS family transcription factors that may regulate MMP9 (Figure S2F). The results showed that the downregulation of the lncRNA STEAP3-AS1 resulted in the most significant decrease in the mRNA and protein levels of ERG (Fig. 2G, Figure S2G). Moreover, we performed rescue experiments and found that when lncRNA STEAP3-AS1 was knocked down or overexpressed, the expression of ERG changed accordingly, indicating that lncRNA STEAP3-AS1 regulates the expression of ERG (Figure S2H). Western blot analyses of eight CRC and normal tissues revealed significantly higher levels of ERG in CRC tissues than in normal tissues (Figure S2I). Therefore, ERG serves as a key transcription factor promoting the ability of the lncRNA STEAP3-AS1 to regulate MMP9.

SWI/SNF subunits are most susceptible to specific inactivating mutations in various cancers. We screened for important core subunits in downstream SWI/SNF complexes regulated by the lncRNA STEAP3-AS1. The results showed that the downregulation of the lncRNA STEAP3-AS1 resulted in the most significant downregulation of BRG1 and P300 (Fig. 2H). After the lncRNA STEAP3-AS1 was overexpressed, the expression of BRG1, ERG, and MMP9 was significantly upregulated (Fig. 2I). We subsequently analysed the expression of BRG1 in the organoids and found that BRG1 was significantly overexpressed in the organoids and CRC tissues (Fig. 2J), whereas the knockdown of BRG1 significantly inhibited the growth of the organoids [26] (Fig. 2K). We also analysed the expression of BRG1 in a CRC liver metastasis model and found that BRG1 expression was inhibited in the liver of the lncRNA STEAP3-AS1-knockdown group (Fig. 2L, Figure S2J). Collectively, these data suggest that the lncRNA STEAP3-AS1 regulates the chromatin remodelling factor BRG1 and the metastasis-related molecule MMP9 to promote CRC liver metastasis.

### The LncRNA STEAP3-AS1 mediates R-loop formation in the BRG1 gene and promotes BRG1 expression

**Fig. 3:**
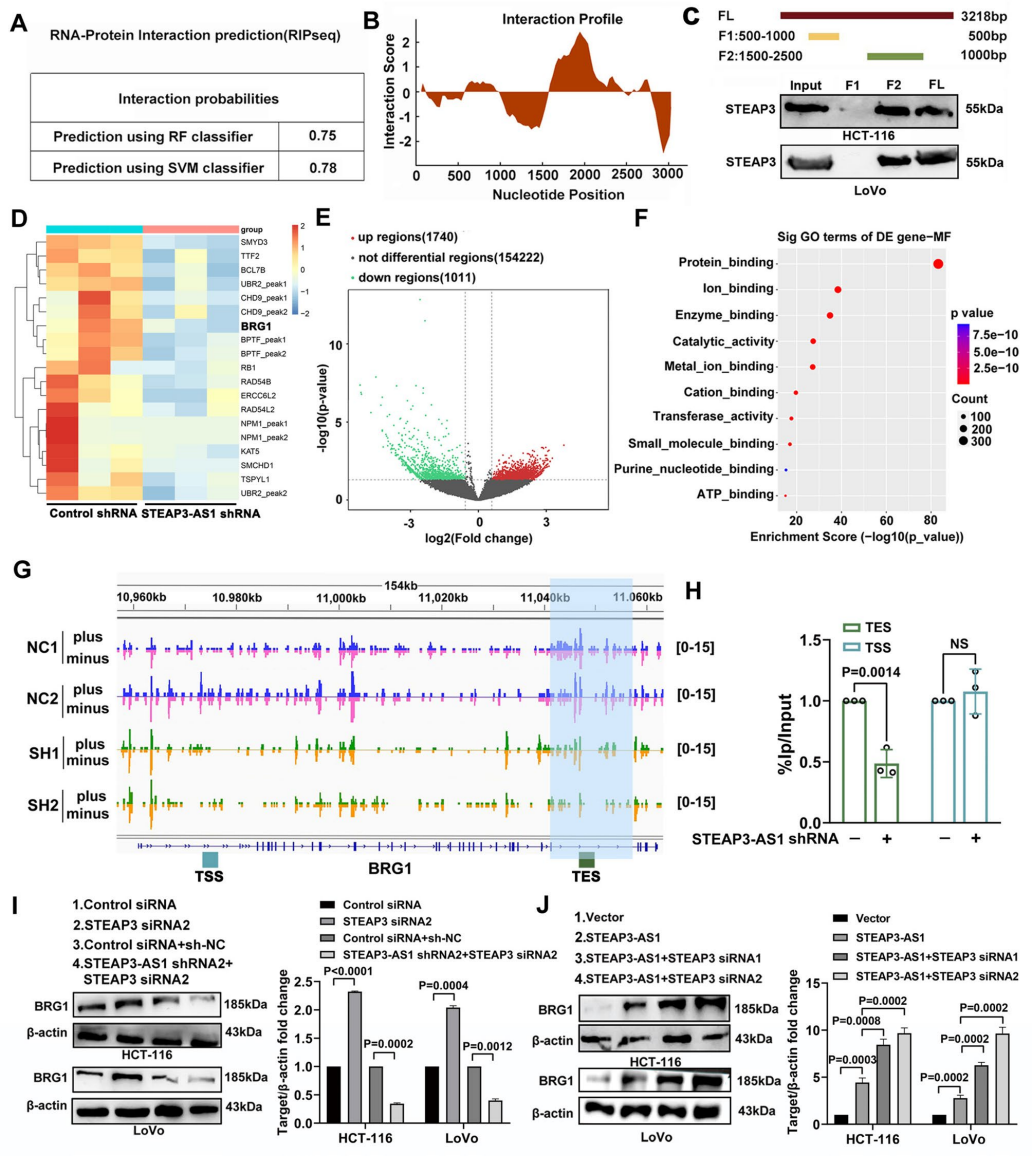
The lncRNA STEAP3-AS1 binds to the STEAP3 protein to regulate BRG1 expression. (**A**) The RPISeq database was used to predict the possibility of an interaction between the lncRNA STEAP3-AS1 and STEAP3. (**B**) Interaction profile of the lncRNA STEAP3-AS1 and STEAP3; the peak value represents the strength of the binding force. (**C**) STEAP3 bound to the lncRNA STEAP3-AS1, as confirmed by Western blot analysis after RNA pull-down. (**D**) Heatmaps showing the expression of genes. *N* = 3 biological replicates. The colour key represents the log2-transformed fold change relative to the mean of each row; blue indicates lower expression levels, and red indicates higher expression levels. (**E**) Volcano plots of differential lncRNA STEAP3-AS1 expression between the control and lncRNA STEAP3-AS1-knockdown groups. (**F**) GO biological process categories enriched in the cluster of genes correlated with the lncRNA STEAP3-AS1 in the DRIP-seq analysis. (**G**) IGV tracks for the differential enrichment of R-loops in the BRG1 gene from the DRIP-seq analysis. (**H**) DRIP‒qPCR was used to analyse the differential enrichment of the R-loop at the BRG1 terminator position. (**I**) Western blot analysis of the expression of BRG1 after the coknockdown of the lncRNA STEAP3-AS1 and STEAP3 in HCT-116 and LoVo cells. (**J**) Western blot analysis of the expression of BRG1 after the overexpression of the lncRNA STEAP3-AS1

Previous studies have shown that strong binding may occur between the lncRNA STEAP3-AS1 and the parental gene STEAP3 [23]. We utilized the RPISeq database for the binding affinity analysis to investigate the interaction between the lncRNA STEAP3-AS1 and the STEAP3 protein. As shown in Fig. 3A, the binding score exceeded 0.5, indicating a strong interaction between the two molecules (Figure S3A). Furthermore, we used the CatRAPID database to predict that strong binding between the 1500–2500 nt region of the lncRNA STEAP3-AS1 and the STEAP3 protein (Fig. 3B). Therefore, we designed biotin-labelled full-length probes and two truncated probes to detect the binding of the lncRNA STEAP3-AS1 to the STEAP3 protein. The results showed that the truncated lncRNA STEAP3-AS1 probe from 1500 to 2500 nt bound to more STEAP3 proteins, whereas the RNA probes from 500 to 1000 nt showed almost negative binding (Fig. 3C). These findings indicate strong binding between the STEAP3 protein and the lncRNA STEAP3-AS1 in the 1500–2500 nt region. Research has shown that the SWI/SNF chromatin remodelling complex helps resolve R-loop-mediated transcription‒replication conflicts and protects genomic integrity [11]. LncRNAs regulate the formation of R-loops at any position in the genome, thereby affecting gene expression [27]. Therefore, we speculate that the lncRNA STEAP3-AS1 affects the chromatin accessibility of genes and is related to the R-loop structure. Next, a DRIP-seq experiment was conducted. The results revealed that the downregulation of the lncRNA STEAP3-AS1 had a significant effect on the R-loop levels of multiple chromatin remodelling genes (Fig. 3D&E, supplement Excel 1). The GO analysis indicated that the R-loops of the differentially expressed genes had protein-binding functions (Fig. 3F). Interestingly, the R-loopBase database (http://rloop.bii.a-star.edu.sg/) revealed that the BRG1 termination region has a high GC skew, and the DRIP-seq results indicated that the lncRNA STEAP3-AS1 significantly affected R-loop enrichment in the BRG1 termination region [28] (Fig. 3G). The R-loop in the gene termination subregion can act as a Pol II promoter to promote gene transcription or recruit the transcription elongation factor PAF1 to prolong transcription [29]. We further analysed the R-loop level of the BRG1 site using the R-loopBase database. Consistent with the above results, BRG1 has a relatively high GC bias in the termination region, indicating a high probability of forming an R-loop structure in this region (Figure S3D). We used DRIP-qPCR to analyse the R-loop of the BRG1 termination region, and the results showed that the lncRNA STEAP3-AS1 affected the R-loop enrichment of the BRG1 termination region (Fig. 3H). We further analysed the regulation of BRG1 by the interaction between the lncRNA STEAP3-AS1 and STEAP3. First, we detected the knockdown efficiency of STEAP3 siRNAs (siRNA1 and siRNA2) in HCT-116 and LoVo cells (Figure S3E-G). After STEAP3 was knocked down, the expression of BRG1 was upregulated (Figure S3H). By knocking down the lncRNA STEAP3-AS1, we found that knocking down STEAP3 together with the lncRNA STEAP3-AS1 significantly reduced the level of the BRG1 protein (Fig. 3I, Figure S3I). However, overexpressing the lncRNA STEAP3-AS1, the expression of BRG1 was upregulated, and after silencing STEAP3, the expression of BRG1 was also upregulated. Therefore, after overexpressing lncRNA STEAP3-AS1 and silencing STEAP3, the upregulation of BRG1 expression was more significant (Fig. 3J). These findings suggest that the interaction between the lncRNA STEAP3-AS1 and STEAP3 positively regulates the expression of BRG1.

### The LncRNA STEAP3-AS1 participates in CRC chromatin remodelling by regulating H3K18la

**Fig. 4:**
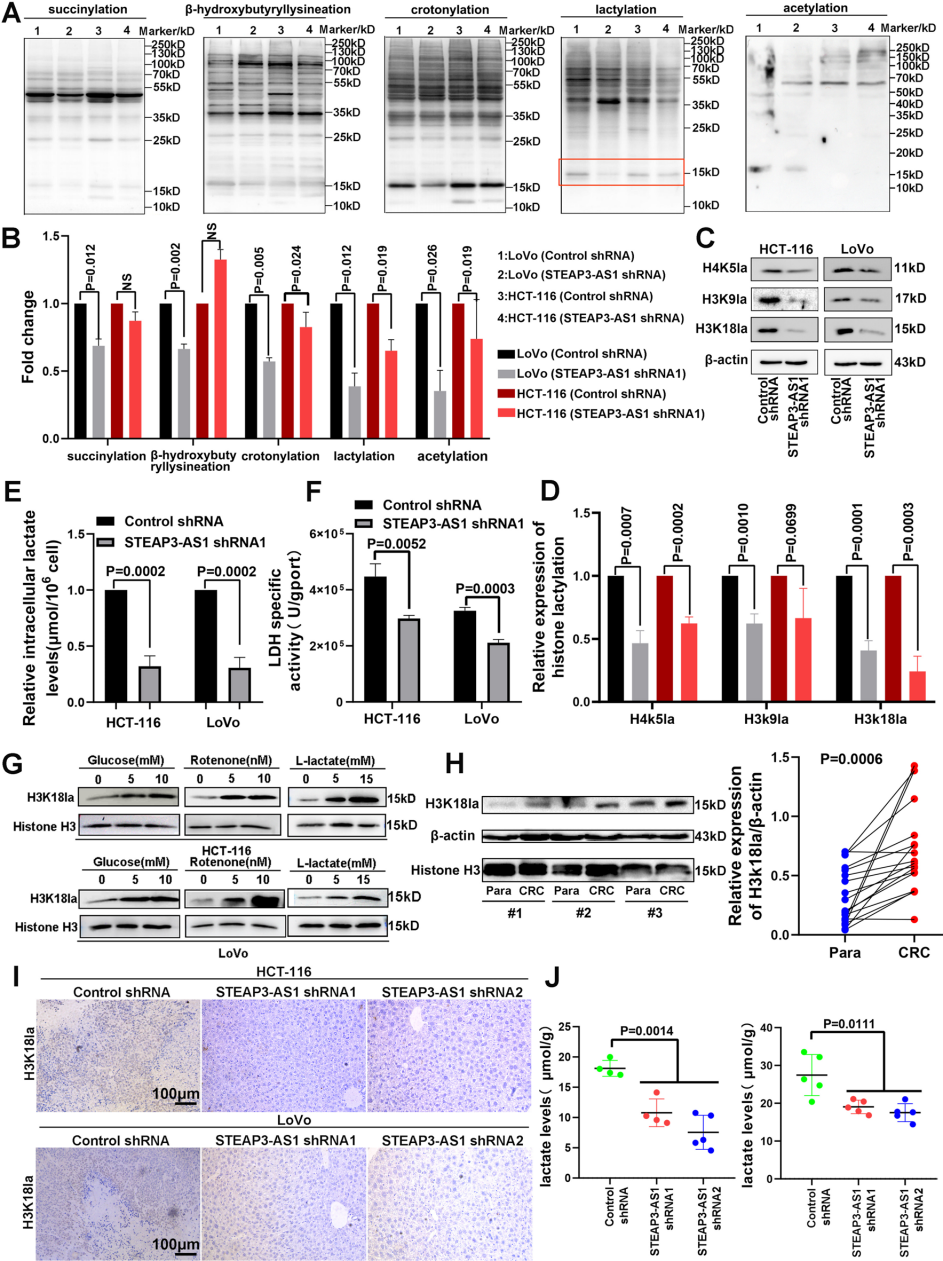
The lncRNA STEAP3-AS1 regulates H3K18la and promotes CRC liver metastasis. (**A** and **B**) Histone Ksucc, Kbhb, Kcr, Kla and Kac levels were analysed by Western blotting of whole-cell lysates from LoVo and HCT-116 cells. (**C** and **D**) Western blot analysis of site-specific histone lactylation in HCT-116 and LoVo cells. (**E** and **F**) Intracellular lactate and LDH-specific activity levels were measured in HCT-116 and LoVo cells. (**G**) Western blot analysis of the level of H3K18la in HCT-116 and LoVo cells treated with different glycolysis modulators (glucose, rotenone and L-lactate). (**H**) Western blot analysis of H3K18la levels in 18 CRC tissues. (**I**) IHC for H3K18la in the liver metastatic tissues of the liver injection model mice. (**J**) Intracellular lactate levels were measured in liver metastatic tissues from the liver injection model mice. Scale bars, 100 μm

Histones undergo specific posttranslational modifications that regulate chromatin structure and function via different mechanisms of action [30]. Research reports indicate that succinylation [31], β-hydroxybutyryllysination [32], crotonylation [33], lactylation [14], and acetylation [34] play important roles in chromatin remodelling. We performed experiments to reveal the important mechanism and regulatory relationship between the lncRNA STEAP3-AS1 and histone modifications in the occurrence and metastasis of CRC. As shown in Fig. 4A and B, knockdown of the lncRNA STEAP3-AS1 significantly decreased the histone lactylation level, whereas the levels of other histone modifications were not significantly altered. Consistently, global and intracellular lactate levels were also increased in most CRC cell lines (Figure S4A and B). These findings suggest that lactylation plays an important role in the occurrence and development of CRC. Next, we explored the specific sites regulated by the lncRNA STEAP3-AS1, and Western blot analysis showed that the downregulation of H3K18la (H3K18la, H4K5la and H3K9la are crucial for gene transcription [14, 35]) in the lncRNA STEAP3-AS1-knockdown group was more significant than that in the control group (Fig. 4C and D, Figure S4C and D).

Accumulated lactate, a biochemical precursor, generates lactyl-CoA through enzymatic reactions in the cell nucleus, thereby promoting histone lactylation [14, 36]. As shown in Fig. 4E and F, knockdown of the lncRNA STEAP3-AS1 significantly reduced the intracellular lactate levels and LDH activity of HCT-116 and LoVo cells. Here, (1) glucose, (2) rotenone, and (3) L-lactate were used to promote lactate production and histone lactylation [14]. Notably, glycolysis significantly increased H3K18la levels in a dose-dependent manner (Fig. 4G) in CRC cells. The results of the CCK-8 assay revealed that L-lactate treatment significantly promoted the proliferation of CRC cells, whereas the downregulation of the lncRNA STEAP3-AS1 significantly reversed cell proliferation compared with that of the negative control (Figure S4E), and the results of the wound healing assay further confirmed this result (Figure S4F and G). Furthermore, transwell migration assays demonstrated that lncRNA STEAP3-AS1 silencing markedly decreased the migratory potential of CRC cells (Figure S4H). Western blot analyses of 18 CRC and normal tissues revealed significantly higher levels of H3K18la in CRC tissues than in normal tissues (Fig. 4H, Figure S4I). We measured intracellular lactate levels in the livers of nude mice to further analyse the role of H3K181a in CRC liver metastasis. The results showed that the H3K18la and intracellular lactate levels were significantly increased in liver metastatic tissue, whereas the H3K18la and intracellular lactate levels were significantly reduced in liver tissue without tumour metastasis in the lncRNA STEAP3-AS1 knockdown group (Fig. 4I and J, Figure S4J and K). These results suggest that the lncRNA STEAP3-AS1 participates in CRC chromatin remodelling by regulating H3K18la levels to promote the liver metastasis of CRC cells.

### The LncRNA STEAP3-AS1 mediates the regulation of H3K18la activation of MMP9 gene expression by the BRG1/ERG/P300 complex

**Fig. 5:**
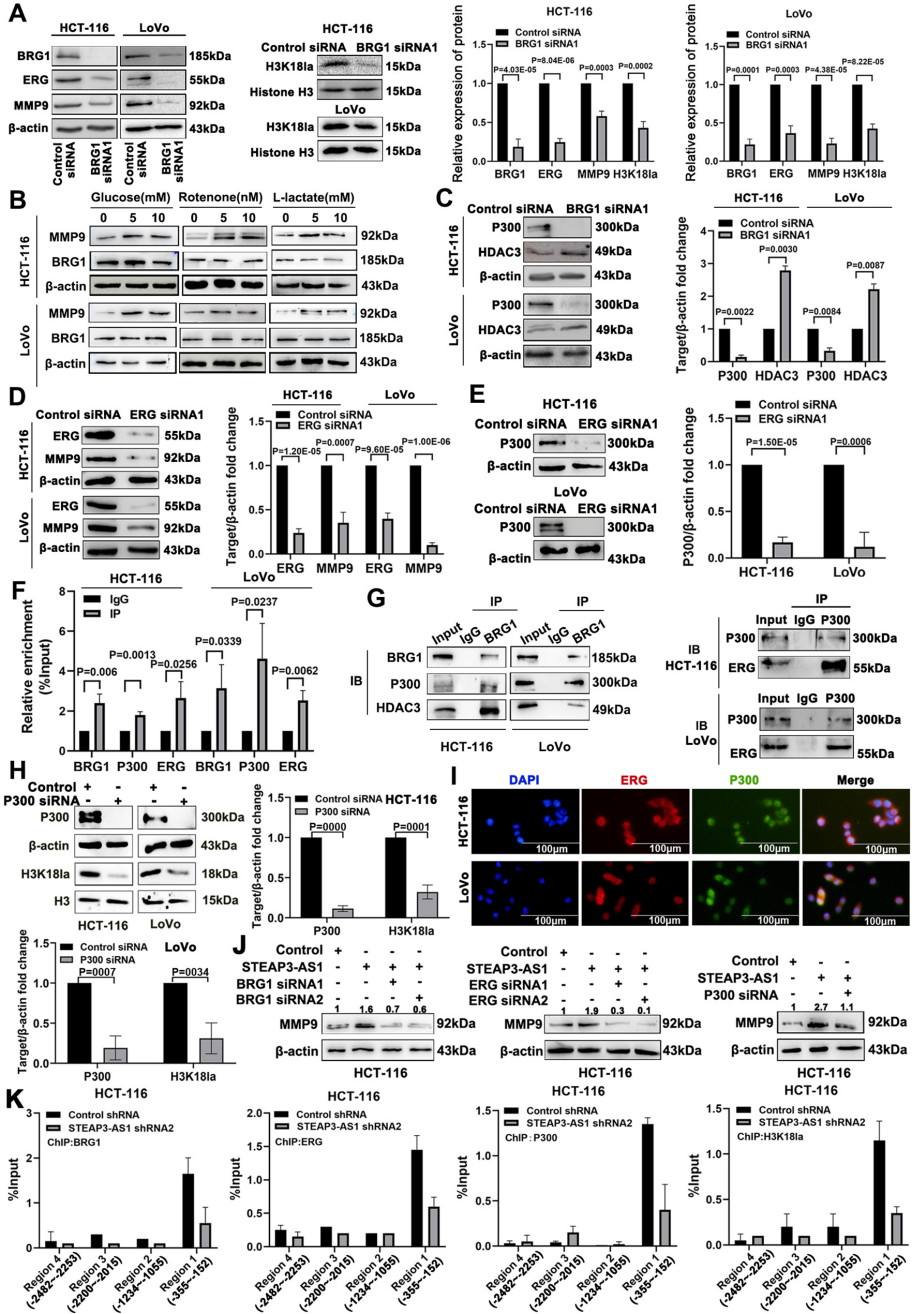
The lncRNA STEAP3-AS1 mediates the regulation of H3K18la activation of MMP9 gene expression by the BRG1/ERG/P300 complex. (**A**) The protein levels of ERG, MMP9 and H3K18la Win HCT-116 cells after transfection with BRG1 siRNA1 were determined by Western blotting. (**B**) Western blotting was performed to assess BRG1 and MMP9 expression in HCT-116 cells. (**C**) Western blotting was performed to detect the levels of the P300 and HDAC3 proteins after BRG1 knockdown. (**D**) Western blots were performed to determine MMP9 protein levels Win CRC cells after transfection with the ERG siRNA1. (**E**) Western blotting was used to detect the P300 protein levels after ERG knockdown. (**F**) A RIP assay was used to detect the enrichment efficiency of BRG1, P300 and ERG at the lncRNA STEAP3-AS1. (**G**) A co-IP assay was performed to detect the interactions between the BRG1, P300, HDAC3 and ERG proteins. (**H**) Western blotting was used to detect H3K18la protein levels after P300 knockdown. (**I**) An immunofluorescence assay was performed to detect the subcellular localization of the ERG and P300 proteins. (**J**) Western blot analysis of the MMP9 protein levels. (**K**) ChIP‒qPCR was used to detect the enrichment efficiency of BRG1, ERG, P300 and H3K18la at four sites in the promoter region of MMP9 in HCT-116 cells

We further investigated the regulatory mechanism of lncRNA STEAP3-AS1-mediated chromatin remodelling and histone modification in the liver metastasis of CRC by assessing the regulatory relationships among BRG1, ERG, H3K18la, and MMP9. BRG1 knockdown significantly reduced the levels of ERG, H3K18la, and MMP9 (Fig. 5A, Figure S5A). Notably, glucose, rotenone and L-lactate caused significant dose-dependent increases in the level of H3K18la, and the MMP9 protein level increased in a dose-dependent manner, while no significant change in BRG1 expression was observed (Fig. 5B). After LDHA and LDHB were inhibited, the levels of H3K18la and MMP9 were significantly decreased, whereas the expression of BRG1 remained unchanged (Figure S5B and C). These results suggest that BRG1 regulates histone lactylation. Studies have shown that H3K18la is deposited or removed by the acetyltransferases CBP/P300 and HDAC3 [37]. Therefore, we explored the molecular mechanism by which BRG1 regulates H3K18la levels. BRG1 knockdown significantly reduced the expression level of P300, whereas the expression level of HDAC3 was significantly increased (Fig. 5C, Figure S5D). We also explored the effect of the transcription factor ERG on MMP9 expression. The expression level of MMP9 decreased significantly after ERG knockdown, whereas the expression level of MMP9 increased in a dose-dependent manner after ERG overexpression (Fig. 5D, Figure S5E and F). Moreover, P300 acts as a coactivator and interacts with ERG to regulate the transcription of target genes [38]. Interestingly, ERG knockdown led to substantially reduced levels of the P300 protein (Fig. 5E, Figure S5G). We speculated that MMP9 expression was activated by the lncRNA STEAP3-AS1-mediated BRG1/ERG/P300 complex during CRC liver metastasis. Using the RIP assay, we found that the lncRNA STEAP3-AS1 was markedly enriched in the pull-down experiments with antibodies against BRG1, ERG, and P300 (Fig. 5F, Figure S5H). Moreover, co-IP experiments confirmed that the BRG1 antibody effectively precipitated P300 and HDAC3 and that the P300 antibody effectively precipitated ERG (Fig. 5G). After P300 expression was downregulated, H3K18la levels were significantly downregulated (Fig. 5H). Notably, P300 downregulation led to a corresponding decrease in the expression of the lncRNA STEAP3-AS1. This observation suggests that P300 may indirectly exert regulatory effects on lncRNA STEAP3-AS1 through the modulation of its parent gene STEAP3 [39] (Figure S5I). The IF results also showed that ERG and P300 colocalized in the nucleus (Fig. 5I, Figure S5J). These results suggest that the lncRNA STEAP3-AS1 mediates the formation of the BRG1/ERG/P300 complex, which regulates histone lactylation and promotes transcriptional activation in a P300-dependent manner.

We detected the expression of MMP9 after the overexpression of the lncRNA STEAP3-AS1 to further validate the effect of lncRNA STEAP3-AS1-mediated BRG1/ERG/P300 complex on promoting MMP9 expression and found that MMP9 protein levels were strikingly increased; this effect was reversed after the silencing of BRG1, EGR, and P300 (Fig. 5J, Figure S5K). We confirmed that MMP9 transcription was activated by the BRG1/ERG/P300 complex by detecting the binding of BRG1, ERG, P300, and H3K18la to the MMP9 promoter. ChIP‒qPCR assays indicated that BRG1, ERG, P300, and H3K18la were enriched at region 1 of the MMP9 promoter and that this enrichment was reduced after the lncRNA STEAP3-AS1 was silenced (Fig. 5K, Figure S5L). These observational results further support the view that the lncRNA STEAP3-AS1 mediates BRG1 recruitment of P300 and HDAC3 to maintain a balance of histone lactylation and maintain a globally active chromatin state, inducing the active transcription of MMP9.

### The LncRNA STEAP3-AS1 interacts with the parental gene STEAP3 to promote the proliferation and migration of CRC cells

**Fig. 6:**
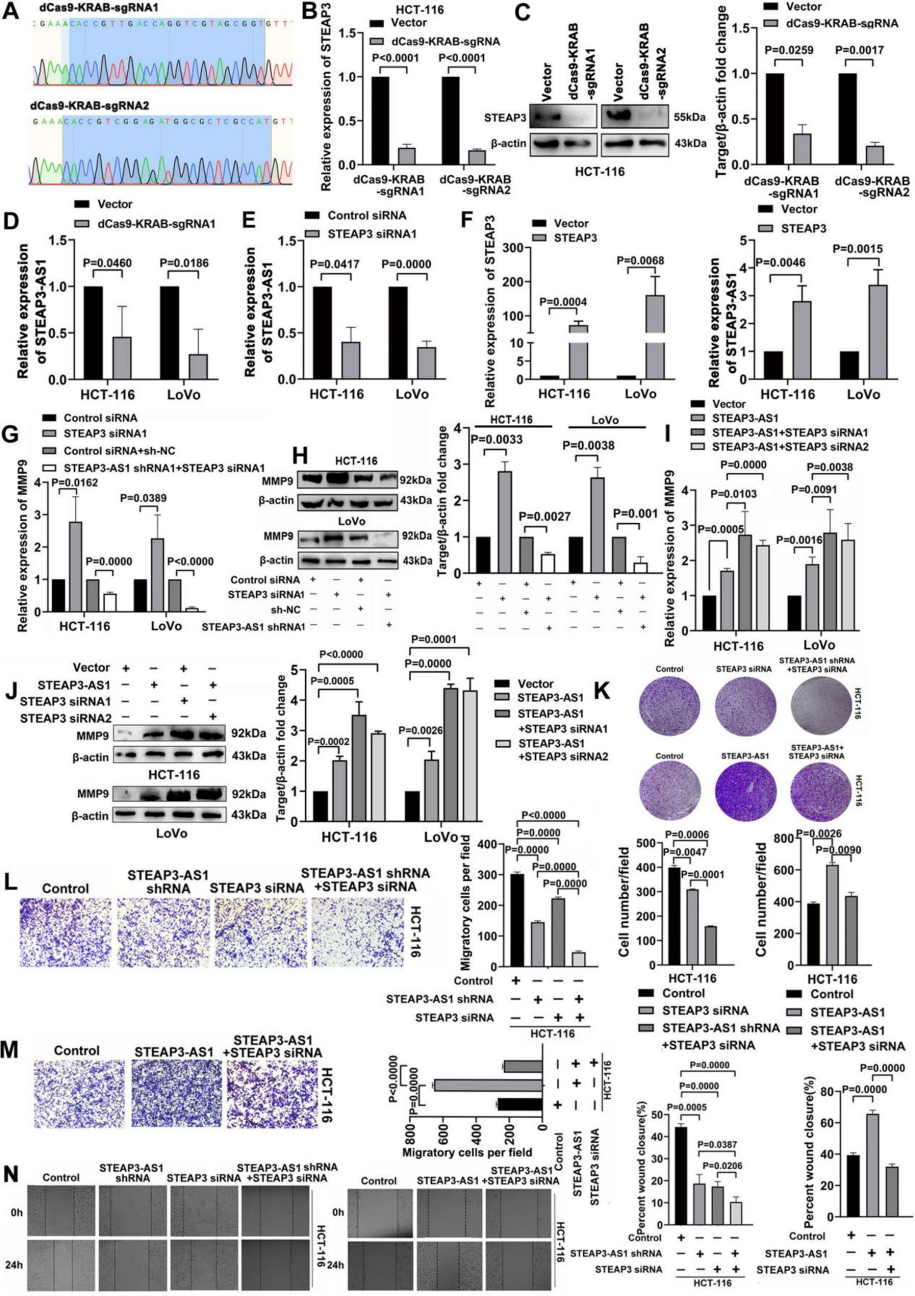
The lncRNA STEAP3-AS1 interacts with the parental gene STEAP3 to promote the proliferation and migration of CRC cells. (**A**) Sanger sequencing was used to identify the sequences of the STEAP3 recombinant plasmids. (**B**-**E**) RT‒qPCR and Western blotting were performed to detect the expression levels of STEAP3 and the lncRNA STEAP3-AS1 in HCT-116 cells after transfection with the STEAP3 sgRNA plasmid and STEAP3 siRNA. (**F**-**J**) RT‒qPCR and Western blotting were performed to detect the expression levels of STEAP3, the lncRNA STEAP3-AS1 and MMP9. (**K**) The proliferation ability of HCT-116 cells was evaluated by colony formation assays. (**L** and **M**) Representative images of the migration of HCT-116 cells on the membrane and statistical analysis. Scale bars, 200 μm. (**N**) The migratory ability of HCT-116 cells was evaluated by wound healing assays. Scale bars, 200 μm

Antisense lncRNAs regulate parental gene expression. Research has shown that the lncRNA SATB2-AS1 promotes SATB2 expression by modulating DNA demethylation and H3K4me3 enrichment at the SATB2 promoter by recruiting the WDR5 and GADD45A proteins [40]. The lncRNA STEAP3-AS1 is located on the antisense strand of the homologous gene STEAP3. Strong binding between the STEAP3 protein and the lncRNA STEAP3-AS1 was observed in the 1500–2500 nt region. We designed a recombinant plasmid for STEAP3 to silence its expression and further investigate the regulatory effect of STEAP3 on the lncRNA STEAP3-AS1 (Fig. 6A, Figure S6A). As shown in Fig. 6B and C, the sgRNA significantly inhibited the expression of STEAP3 (Figure S6B and C). After the decrease in STEAP3 expression, the expression of the lncRNA STEAP3-AS1 was significantly inhibited (Fig. 6D and E, Figure S6D and E). We overexpressed STEAP3 and found that the expression of the lncRNA STEAP3-AS1 was significantly upregulated (Fig. 6F). These findings suggest that the parental gene STEAP3 positively regulates the expression of the lncRNA STEAP3-AS1 and plays a biological role.

In this study, we found that the lncRNA STEAP3-AS1 interacts with STEAP3 to form an R-loop that regulates the expression of the important chromatin remodelling factor BRG1. Therefore, we speculated that the interaction between the lncRNA STEAP3-AS1 and STEAP3 regulates the expression of the target gene MMP9, promoting the liver metastasis of CRC. The results showed that STEAP3 knockdown upregulated the expression of MMP9, whereas the knockdown of lncRNA STEAP3-AS1 together with STEAP3 significantly downregulated the expression of MMP9 (Fig. 6G and H, Figure S6F and G), indicating that the lncRNA STEAP3-AS1 mainly promotes CRC liver metastasis by interacting with STEAP3 to regulate MMP9 expression. As expected, the overexpression of the lncRNA STEAP3-AS1 and the knockdown of STEAP3 increased the expression of MMP9 (Fig. 6I and J). In addition, we observed that the knockdown of lncRNA STEAP3-AS1 together with STEAP3 significantly inhibited the proliferation and migration of colon cancer cells (Fig. 6K and L, Figure S6H and I), whereas the overexpression of the lncRNA STEAP3-AS1 significantly promoted the proliferation and migration of colon cancer cells (Fig. 6M and N, Figure S6J and K). In summary, these results indicate that the parental gene STEAP3 positively regulates the expression of the lncRNA STEAP3-AS1 in colorectal cancer and that the interaction between the lncRNA STEAP3-AS1 and STEAP3 is involved in chromatin remodelling of the target gene MMP9, thereby promoting the liver metastasis of CRC.

### The expression of the LncRNA STEAP3-AS1 is positively correlated with BRG1 and H3K18la levels in human CRC samples

**Fig. 7:**
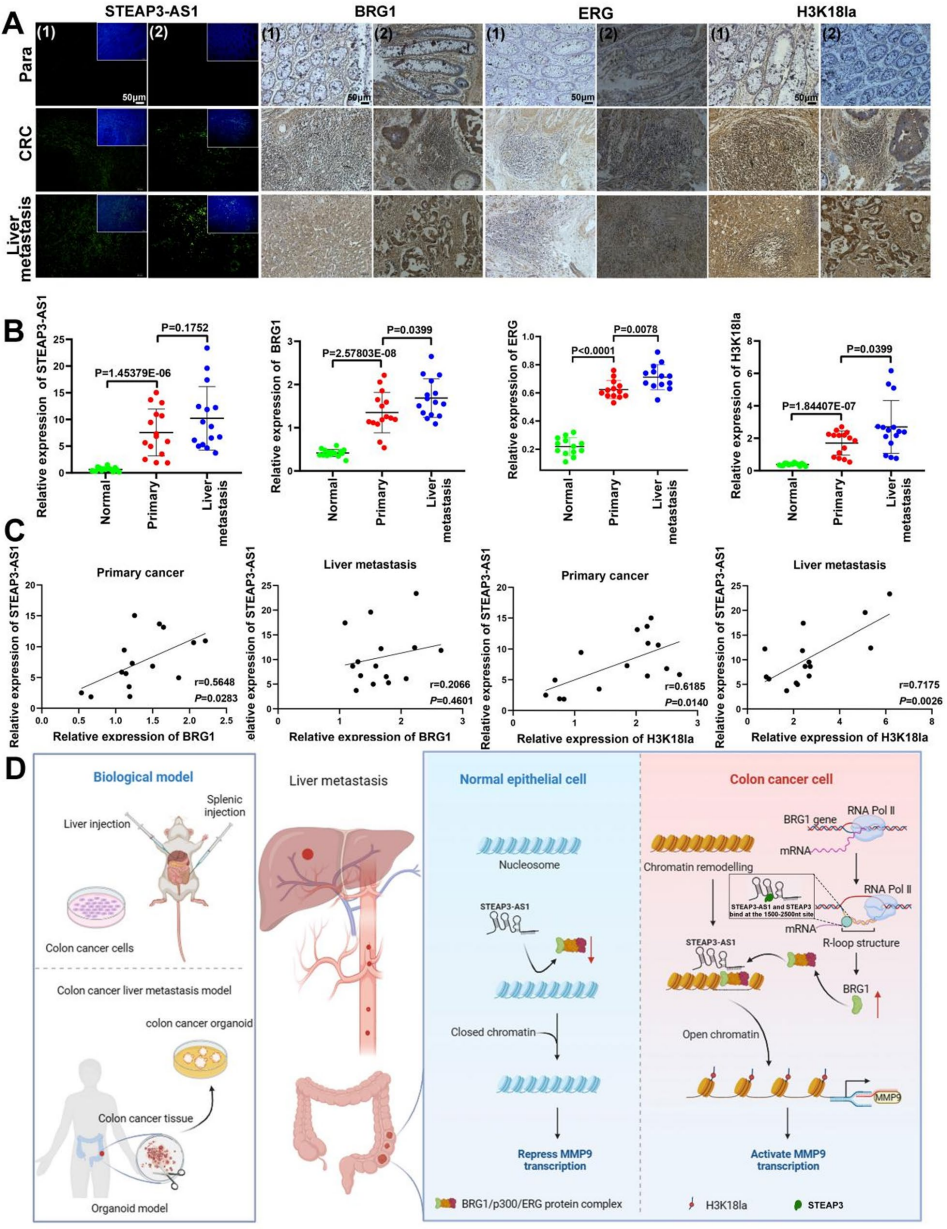
The lncRNA STEAP3-AS1 promotes CRC metastasis through the BRG1-mediated regulation of H3K18la-mediated chromatin remodelling. (**A** and **B**) FISH and IHC detection of the lncRNA STEAP3-AS1, BRG1, H3K18la and ERG expression levels in clinical tissues and statistical analysis. (**C**) Correlations between lncRNA STEAP3-AS1, BRG1 or H3K18la expression in primary CRC and liver metastasis. (**D**) Schematic diagram of the mechanism by which the lncRNA STEAP3-AS1 promotes CRC metastasis through BRG1-mediated regulation of chromatin remodelling through H3K18la

We determined potential correlations between the lncRNA STEAP3-AS1, BRG1, and H3K18la levels in primary CRC and liver metastasis to further analyse the clinical relevance of our results. We analysed 15 matched normal, primary CRC, and liver metastasis samples. FISH and IHC staining revealed that the expression of the lncRNA STEAP3-AS1, BRG1, ERG, and H3K18la was increased in liver metastases than in their corresponding primary CRC samples (Fig. 7A and B). An analysis of the matched 54 normal, 186 primary CRC, and 47 liver metastatic samples from GEO (GSE41258) confirmed that STEAP3, BRG1, ERG and P300 were upregulated in liver metastatic samples (Figure S7A). In addition, a direct correlation was observed between the lncRNA STEAP3-AS1, BRG1, and H3K18la expression levels in both primary cancer and liver metastatic tissues (Fig. 7C). These clinical data support our preclinical findings and suggest that the upregulation of the lncRNA STEAP3-AS1 occurs in the setting of liver metastasis in patients with CRC, and this upregulation may be consistent with chromatin remodelling disorders in CRC (Fig. 7D).

## Discussion

CRC is one of the main causes of cancer-related death worldwide, and increasing evidence suggests that epigenetic changes caused by the dysregulation of epigenetic modifying factors play crucial roles in the occurrence, development, and metastasis of CRC [41]. Chromatin remodelling and histone modification are considered the main processes that control the transcription of oncogenes and affect the occurrence and development of CRC [42]. In this study, we first explored the potential role of the lncRNA STEAP3-AS1 in regulating histone lactylation through chromatin remodelling in CRC liver metastasis. This lncRNA STEAP3-AS1-mediated regulation of epigenetics represents a promising research strategy for colorectal cancer liver metastasis. In summary, our study provides critical insights into the role of histone lactylation in CRC liver metastasis, emphasizing potential therapeutic targets for this challenging disease.

The dysfunction or abnormal expression of lncRNAs is closely related to tumour processes. LncRNAs regulate the occurrence and development of various tumours by regulating genomic stability, the cell cycle, and metastasis [43]. Studies have shown that the m6A modification promotes the upregulation of the lncRNA BLACAT3 in bladder cancer through a stable RNA structure. The lncRNA BLACAT3 recruits Y-box binding protein 3 (YBX3) into the nucleus, synergistically increases NCF2 transcription and promotes bladder cancer angiogenesis and blood metastasis by activating the downstream NF-κB signalling pathway [44]. Fang X et al. found that the lncRNA PCAT1 is abnormally highly expressed in the exosomes of colorectal cancer cells and that the lncRNA PCAT1 can promote the occurrence of the epithelial–mesenchymal transition (EMT) in circulating tumour cells by regulating the miR-329-3p/netrin-1 axis to ultimately promote colorectal cancer liver metastasis progression [45]. These findings suggest that lncRNAs play important roles in tumour metastasis. Zhou L et al. demonstrated that the hypoxia-induced lncRNA STEAP3-AS1, which is highly expressed in clinical CRC tissues, is induced by HIF-1α-mediated transcriptional activation [46]. This study also revealed that the lncRNA STEAP3-AS1 was significantly upregulated in 15 cases of colorectal cancer (CRC) liver metastases, suggesting its potential as a diagnostic biomarker for metastatic CRC. Functional assays indicated that the lncRNA STEAP3-AS1 promotes liver metastasis in vivo and enhances organoid proliferation in vitro. This effect is related to lncRNA STEAP3-AS1 facilitating MMP9 accessibility and modulating CDKN1C expression, thereby accelerating cell cycle progression in CRC [23].

Chromatin remodelling is a key factor controlling gene expression. A previous study revealed that BRG1, a key factor for chromatin remodelling, is highly expressed in lung cancer, colon cancer, breast cancer, and glioma and promotes the occurrence and development of tumours [47, 48]. Boonsanay et al. observed that the loss of Suv4-20h2-mediated H4K20me3 appears predominantly in right-sided colon tumours, resulting in decreased chromatin remodelling and the derepression of tumour-promoting genes, thereby promoting cancer progression [47]. In this study, we found that BRG1 was positively correlated with the expression of the lncRNA STEAP3-AS1 in primary CRC and liver metastasis, suggesting that the lncRNA STEAP3-AS1 regulates the involvement of BRG1 in CRC liver metastasis. Studies have shown that BRG1-P300 complexes are accompanied by poly ADP-ribose polymerase 1 (PARP1), which is codistributed at the promoters of genes such as CDK4, LIG1, or NEIL3 to ensure an open chromatin structure that allows a low histone density, which is responsible for cancer cell growth and the elimination of DNA damage [48]. In the present study, we found that BRG1 interacts with the histone lactylation eraser HDAC3 and the histone lactylation writer P300 to form protein complexes that regulate the expression of H3K18la. BRG1 and H3K18la bind to region 1 in the MMP9 promoter, promoting chromatin accessibility at the MMP9 gene to maintain transcriptional activity, which is consistent with observations by Sobczak et al. [49]. Hu et al. noted that BRG1, as a reader of H3K18la, specifically recognizes H3K18la and thereby activates the transcription of the target gene. Our results also support this finding. These findings indicate that the lncRNA STEAP3-AS1 mediates the specific recognition of H3K18la by BRG1, which is a key mechanism that promotes the liver metastasis of colorectal cancer [49]. More open chromatin has previously been shown to favour the formation of the R-loop by increasing the chances of nascent DNA‒RNA hybridization [50]. Boonsanay et al. reported that a loss of chromatin compaction in Suv4-20h2-depleted murine colon organoids resulted in an increase in the number of R-loops [47]. In this study, we found that the lncRNA STEAP3-AS1 interacts with its parental gene STEAP3 to form an R-loop on the BRG1 gene, regulating the expression of BRG1.

Histone lactylation has been identified as an epigenetic modification. Histone lactylation directly stimulates gene transcription in chromatin. Research by Yu et al. indicated that histone lactation is significantly increased in ocular melanoma, promoting tumorigenesis by inducing the expression of YTHDF2 [15]. In this study, we showed that H3K18la levels were significantly increased in CRC and paired liver metastatic tissues and were positively correlated with the expression of the lncRNA STEAP3-AS1. Animal experiments further confirmed that the knockdown of the lncRNA STEAP3-AS1 markedly reduced H3K18la levels in metastatic tumours. Notably, we observed a distinct disparity in cell size between normal liver tissue and hepatic metastatic lesions, and further analysis revealed that this discrepancy arose from inherent differences between normal hepatocytes and metastatic colorectal cancer cells in the liver. This phenomenon aligns with previous reports by Gao et al. and Liang et al., in which cancer cells exhibited significant morphological alterations compared with their normal counterparts [6, 51]. These variations may be attributed to the accelerated proliferation of malignant cells and their concomitant loss of specialized physiological functions.

Studies have shown that during the polarization process of M1 macrophages caused by infection, the increase in histone lactylation at the promoter region induces the expression of homeostasis-related genes, including Arg1 [14]. Li et al. found in their study on somatic reprogramming that H3K18la was enriched at pluripotent gene loci, thereby promoting the reprogramming of mouse embryonic fibroblasts [52]. The results of this study also indicate that the use of glucose, rotenone, and L-lactate to promote H3K18la significantly activated MMP9 expression. Galle et al. reported that H3K18la distinctively marks active enhancers that do not overlap with promoter regions (~ dELS), many of which are marked by H3K27ac [53]. Despite their overall genomic similarity, H3K27ac and H3K18la profiles also clearly differ; H3K18la is found at more putative enhancers (dELSs) than H3K27ac [53]. Studies by Li et al. showed that CRC patients who are resistant to bevacizumab treatment present with elevated levels of histone lactylation [54]. The inhibition of histone lactylation can improve the clinical efficacy of bevacizumab in colorectal cancer and effectively inhibit CRC tumorigenesis, progression and survival under hypoxic conditions [54]. In our study, the transcription factor ERG that regulates MMP9 formed a protein complex with P300, and the ChIP‒qPCR results further demonstrated that BRG1, ERG, P300, and H3K18la significantly bound to region 1 of the MMP9 promoter. However, the knockdown of the lncRNA STEAP3-AS1 significantly reduced the binding of BRG1, ERG, P300, and H3K18la, indicating that the lncRNA STEAP3-AS1-mediated BRG1/ERG/P300 complex regulates the H3K18la-specific activation of MMP9, which is a key to colorectal cancer liver metastasis. This study reveals a specific epigenetic mechanism driving colorectal cancer liver metastasis and identifies histone lactylation as a promising therapeutic target for clinical intervention.

## Conclusions

In this study, our findings provide a molecular framework for understanding chromatin remodelling by activating MMP9 to promote CRC liver metastasis. We propose that the lncRNA STEAP3-AS1 mediates chromatin remodelling and regulates H3K18la to promote CRC metastasis. CRC liver metastasis is generally believed to be due to the mechanism of blood transmission. Most of the blood in the colorectum flows back into the liver through the portal vein system, and thus circulating tumour cells in CRC invade the liver with the flow of blood. Borrelli et al. found that a reversal of the epithelial‒mesenchymal transition (EMT) occurs during CRC liver metastasis, promoting the formation of a premetastatic niche and facilitating the targeted liver metastasis of colorectal cancer [55]. The lncRNA STEAP3-AS1 mediates the liver metastasis of CRC, but whether the lncRNA STEAP3-AS1 mediates the formation of the premetastatic niche is our next focus of research.

## Electronic supplementary material

Below is the link to the electronic supplementary material.

Supplementary Material 1

Supplementary Material 2

Supplementary Material 3

## Data Availability

No datasets were generated or analysed during the current study.

## Abbreviations

ARID1a: AT-rich interactive domain 1
ATAC-seq: Assay for Targeting Accessible-Chromatin with high-throughout sequencing
BRG1: Brahma-related gene 1
ChIP: Chromatin Immunoprecipitation
Co-IP: Co-Immunoprecipitation
CRC: Colorectal cancer
DRIP: DNA-RNA immunoprecipitation
ERG: ETS transcription factor ERG
FISH: Fluorescence in situ hybridization
H3K18la: H3 lysine 18 lactylation
H3K9la: H3 lysine 9 lactylation
H4K5la: H4 lysine 5 lactylation
HDAC3: Histone deacetylase 3
IF: Immunofluorescence
MMP9: Matrix metallopeptidase 9
P300: E1A Binding Protein P300
PDO: Patient-derived organoids
qPCR: Quantitative reverse transcription PCR
RIP: RNA Immunoprecipitation
RNA-seq: RNA sequencing
shRNA: Short hairpin RNA
SMARCD1: SWI/SNF related, matrix associated, actin dependent regulator of chromatin, subfamily d, member 1
STEAP3: Six-transmembrane epithelial antigen of the prostate 3
