# Supplementary material for: The LncRNA STEAP3-AS1 promotes liver metastasis in colorectal cancer by regulating histone lactylation through chromatin remodelling
Source: J Exp Clin Cancer Res. 2025 Jul 15;44:205. doi: 10.1186/s13046-025-03461-0 (PMC12261760; doi:10.1186/s13046-025-03461-0)
Supplement: Supplementary file 1 — Supplementary Material 1 [file 13046_2025_3461_MOESM1_ESM.doc]

**Supporting Information**

**LncRNA STEAP3-AS1 promotes liver metastasis in colorectal cancer by regulating histone lactylation through chromatin remodeling**

Jinjuan Lv^2,3^**^†^**, Xiaoqi Yu^2^**^†^**, Xiaoqian Liu^2^**^†^**, Qianshi Zhang^1^**^†^**, Mengyan Zhang^2^, Jianfeng Gao^1^, zhiwei Sun^1^, Feifan Zhang^1^, Yunfei Zuo^2^*, Shuangyi Ren^1^*

**Author Affiliations:**

^1^Departments of Gastrointestinal Surgery, The Second Affifiliated Hospital of Dalian Medical University, Dalian 116023, China

^2^Department of Clinical Biochemistry, College of Laboratory Diagnostic Medicine, Dalian Medical University, Dalian 116044, China

^3^Department of Clinical Laboratory Medicine, Xijing Hospital, Fourth Military Medical University, Xi'an, China.

***Correspondence to:**

Shuangyi Ren, Department of General Surgery, the Second Hospital of Dalian Medical University, Dalian, 116023, China. Phone: 86-411-84675130; Email: [rsydl@aliyun.com](mailto:rsydl@aliyun.com).

Yunfei Zuo, Department of Clinical Biochemistry, Dalian Medical University, Dalian, 116044, China. Phone: 86-411-86110387; Fax: 86-411-86110392; Email: [zyf04112002@aliyun.com](mailto:zyf04112002@aliyun.com)

†These authors have contributed equally to this work and share first authorship

**This file includes:**

**Supplementary Figures and Figure legends**

**Supplementary Materials and Methods**

**Supplementary Tables**

**Supplementary Figures and Figure legends**





**Figure S1. Related to Figure 1.** LncRNA STEAP3-AS1 silencing inhibits the growth of organoids and the liver metastasis of CRC. (A) Analysis of the expression of the lncRNA STEAP3-AS1 in paired CRC and paracancerous tissues using the GSE109454 microarray in the GEO database, N=6. (B) The LncATLAS database was used to predict the subcellular localization of the lncRNA STEAP3-AS1. (C) The effect of lncRNA STEAP3-AS1 overexpression on the invasiveness of CRC cells was evaluated using Transwell assays. Scale bars, 100 μm. (D and E) The effects of lncRNA STEAP3-AS1 overexpression on the proliferation of CRC cells were evaluated by performing colony formation assays (D) and CCK-8 assays (E). (F and G) The effect of lncRNA STEAP3-AS1 overexpression on the migratory ability of CRC cells was evaluated by wound healing assays. Scale bars, 200 μm. (H) Successful cultivation of CRC organoids in vitro. Representative images are shown. Scale bar of the bright field images, 200 μm; scale bar of images of IF staining, 100 μm. (I and J) IHC and IF analyses of the expression of Ki-67 or CK20 in normal and CRC tissues and organoids. Scale bars for IHC, 100 μm; scale bars for IF staining, 50 μm. (K) Bright-field imaging of organoid growth after control or lncRNA STEAP3-AS1 shRNA2 silencing and statistical analysis. RT‒qPCR was used to detect the expression of the lncRNA STEAP3-AS1 in the organoids. The inserts show high-magnification images of a representative organoid. Scale bars, 500 μm. (L) IF staining for Ki-67 in the CRC organoids of the control and lncRNA STEAP3-AS1 shRNA2-silenced groups. DAPI (blue), Ki-67 (red). Scale bars, 100 μm. (M) RT‒qPCR was performed to detect the expression levels of the lncRNA STEAP3-AS1 after transfection with the lncRNA STEAP3-AS1 sgRNA plasmid. (N and O) The effects of the lncRNA STEAP3-AS1 KO on the proliferation of CRC cells were evaluated by colony formation (M) and CCK-8 (N) assays. (P) IHC staining for Ki-67, CK20 and CK7 in the liver metastatic tissue of the liver injection model mice and the splenic injection model mice. Scale bars, 100 μm.


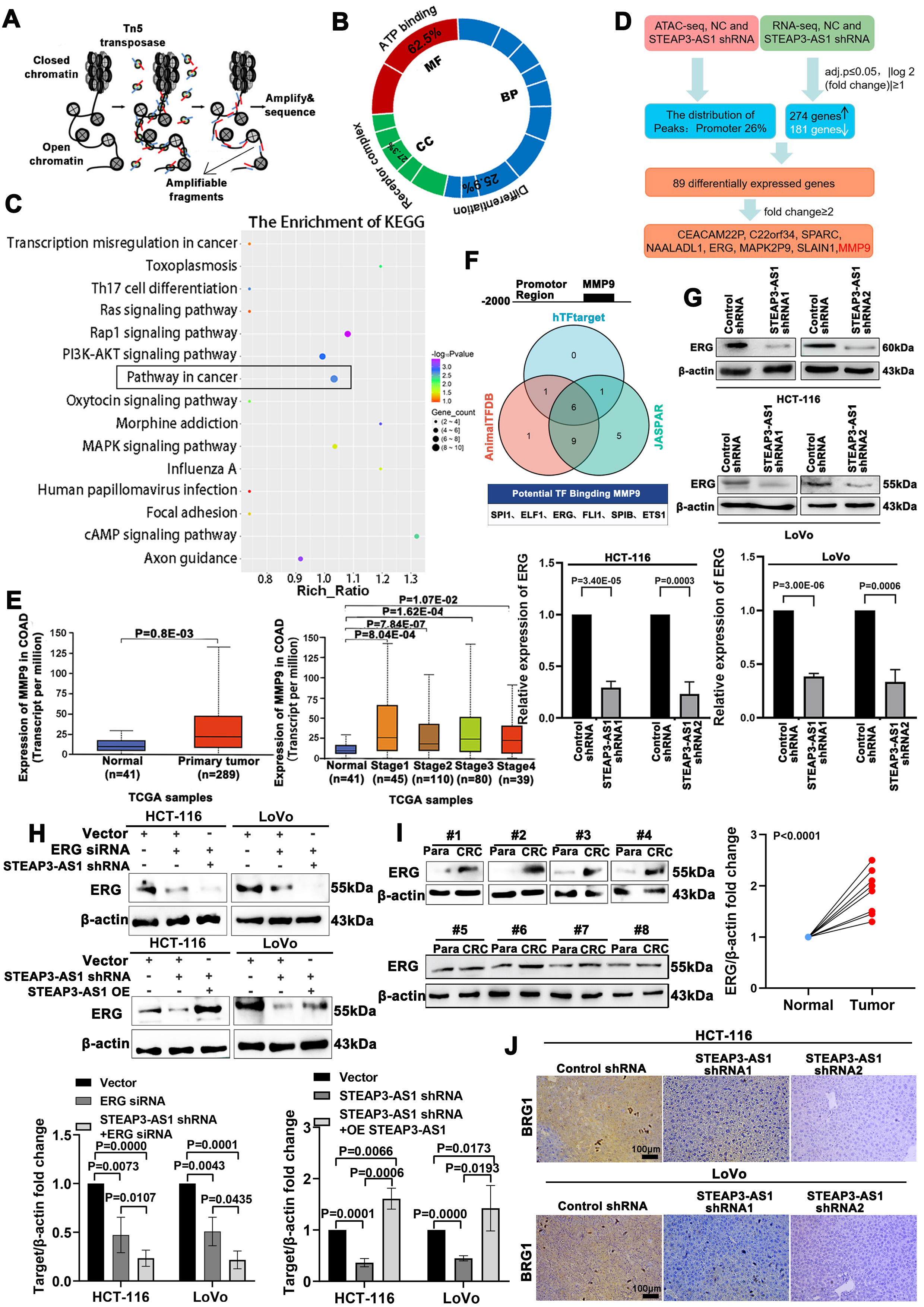


**Figure S****2. Related to Figure 2.** Combined ATAC-seq and RNA-seq analyses of downstream regulatory molecules of the lncRNA STEAP3-AS1. (A) Schematic diagram of the ATAC-seq analysis. (B and C) GO analysis of the biological process category (B) and KEGG enrichment analysis (C) of the cluster of genes correlated with the lncRNA STEAP3-AS1 lncRNA according to RNA-seq and ATAC-seq. (D) Combined ATAC-seq and RNA-seq analyses identified 8 molecules as downstream targets of the lncRNA STEAP3-AS1. (E) TCGA data showing the expression level of MMP9 in CRC tissues or tumours at different stages and adjacent normal tissues. (F) The ETS family of transcription factors that bind to the MMP9 promoter were predicted through the hTFtarget database, AnimalTFBD database, and Jaspar database. (G) Western blot analysis to verify the expression of ERG after the knockdown of the lncRNA STEAP3-AS1; the statistical results of grey value analysis are shown below. (H) The rescue experiment analysed the regulatory effect of lncRNA STEAP3-AS1 on ERG, and the statistical results of the grey value analysis are shown below. (I) Western blot analysis of ERG expression in 8 CRC tissues. (J) IHC staining for BRG1 in the liver metastatic tissue of the splenic injection model mice. Scale bars, 100 μm.
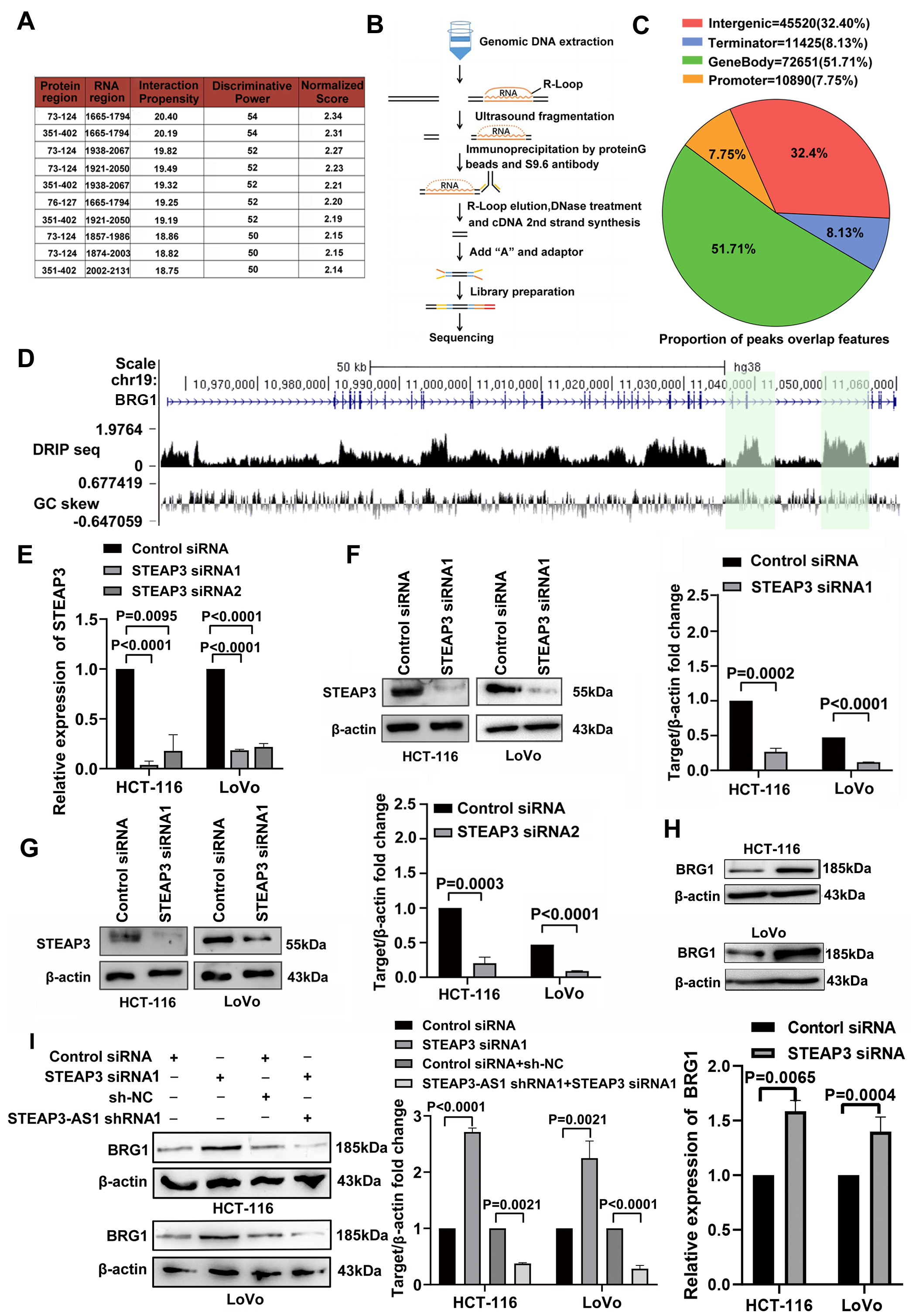


**Figure S3. Related to Figure 3.** The lncRNA STEAP3-AS1 mediates R-loop formation at the termination site of BRG1 and regulates the expression of BRG1. (A) The catRAPID database was used to predict the binding sites of the lncRNA STEAP3-AS1 and STEAP3 gene. (B) Schematic diagram of the DRIP sequencing process. (C) R-loop in the genome-wide distribution map. (D) R-loopBase database-predicted R-loop enrichment in the BRG1 gene. (E) RT‒qPCR was used to detect the expression of STEAP3 in HCT-116 and LoVo cells. (F and G) Western blotting was performed to detect the expression of STEAP3 in HCT-116 and LoVo cells. (H) Western blot analysis of the expression of BRG1 after STEAP3 was knocked down in HCT-116 and LoVo cells. (I) Western blot analysis of the expression of BRG1 after the coknockdown of the lncRNA STEAP3-AS1 and STEAP3 in HCT-116 and LoVo cells.


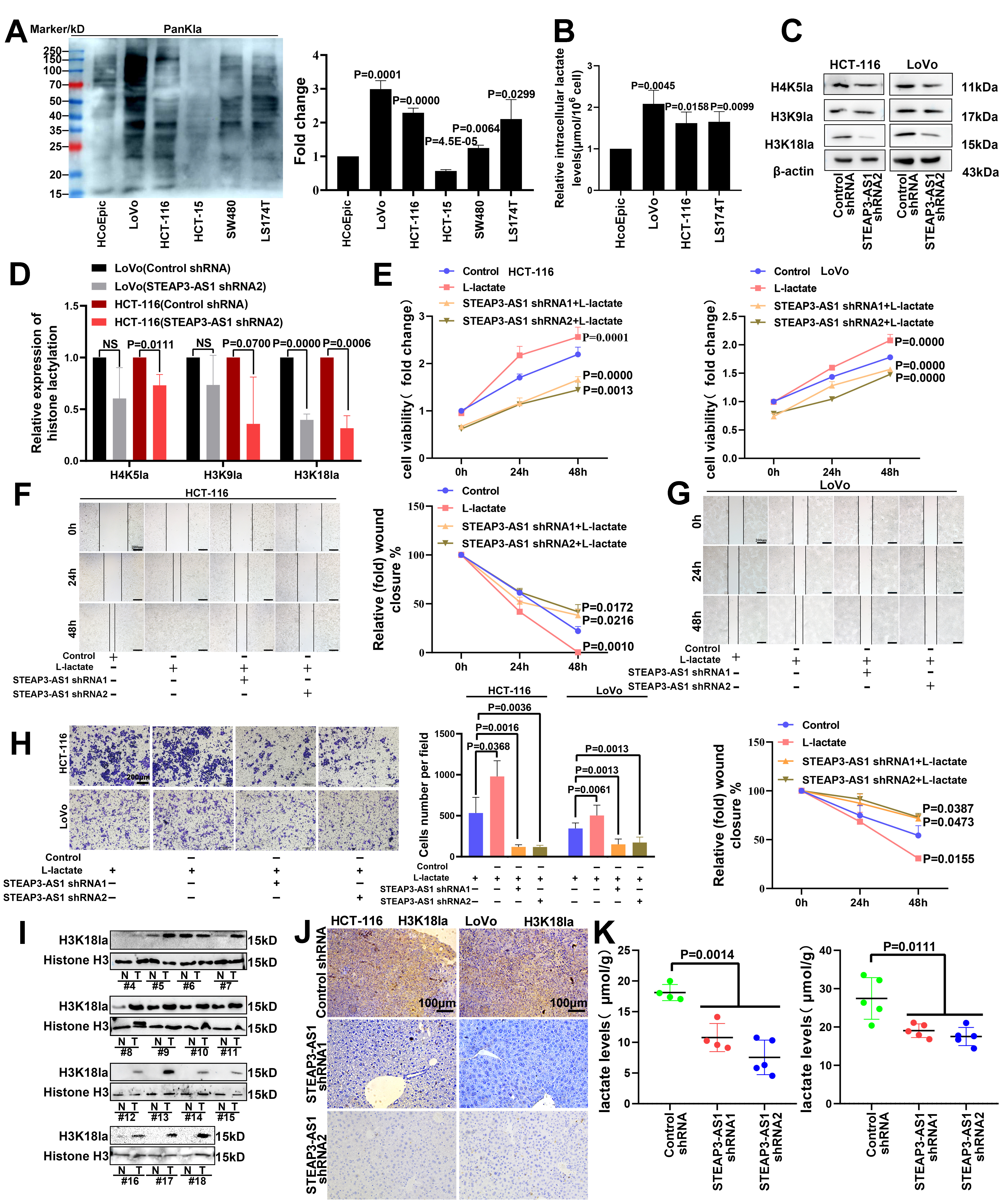


**Figure S4. Related to Figure 4.** H3K18la promotes the growth and metastasis of CRC cells in vitro and in vivo. (A) Histone lactylation levels were detected in HCoEpiC cells and CRC cell lines by Western blotting. (B) Intracellular lactate levels were measured in LoVo, HCT-116 and LS174T cells. (C and D) Western blot analysis of site-specific histone lactylation in HCT-116 and LoVo cells and statistical analysis. (E) A CCK-8 assay was performed to detect the proliferation of HCT-116 and LoVo cells after treatment with L-lactate and lncRNA STEAP3-AS1 silencing. (F and G) The migratory ability of HCT-116 (F) and LoVo (G) cells subjected to L-lactate treatment and lncRNA STEAP3-AS1 silencing was evaluated via a wound healing assay. Scale bars, 200 μm. (H) Representative images of the migration of HCT-116 and LoVo cells on the membrane and statistical analysis. Scale bars, 200 μm. (I) Western blot analysis of H3K18la levels in CRC tissues. (J) IHC staining for H3K18la in the liver metastatic tissue of the splenic injection model mice. (K) Intracellular lactate levels were measured in liver metastatic tissue from the splenic injection model mice.


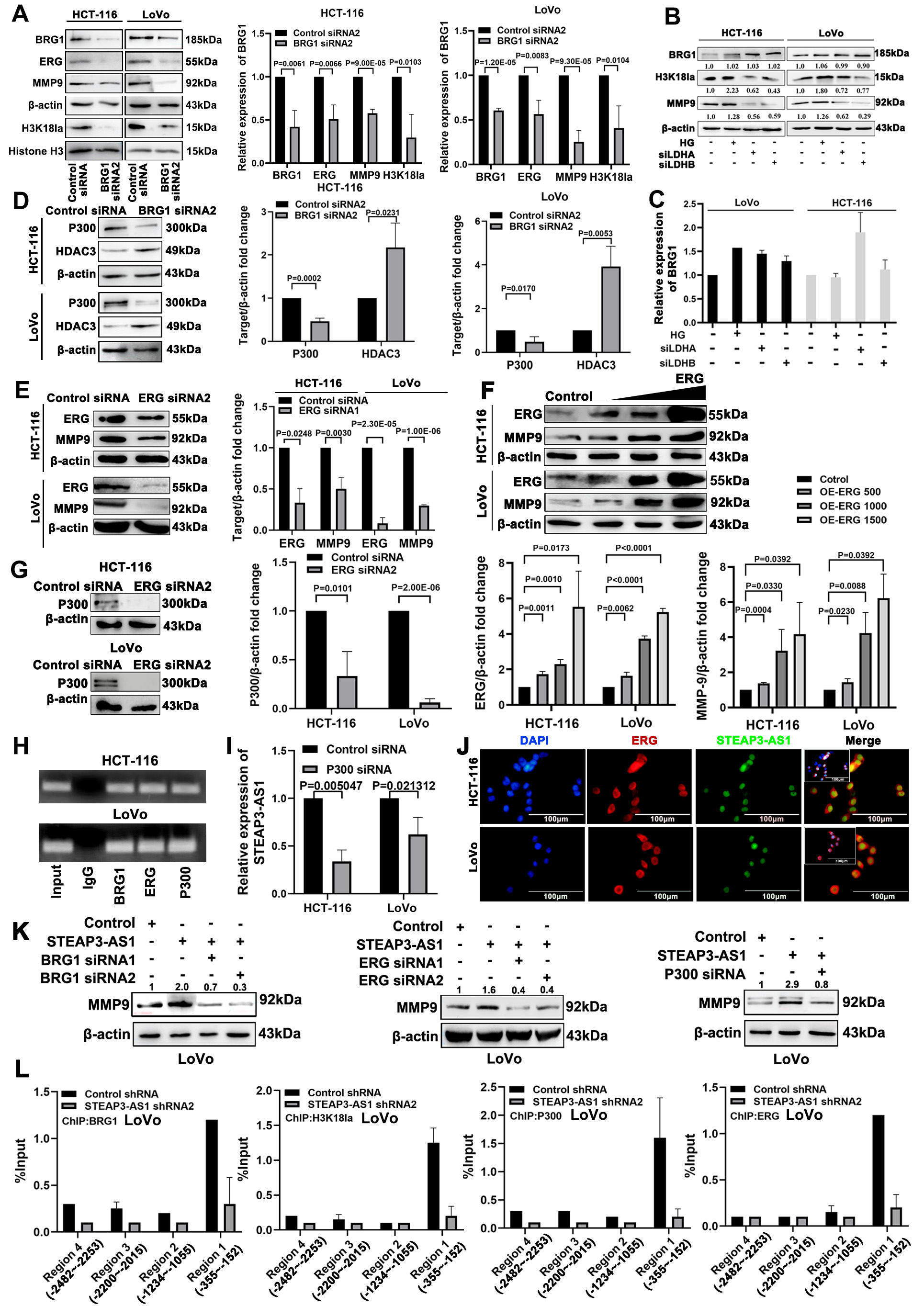


**Figure S5. Related to Figure 5.** The lncRNA STEAP3-AS1 mediates the regulation of H3K18la activation of MMP9 gene expression by the BRG1/ERG/P300 complex. (A) The protein levels of ERG, MMP9 and H3K18la in CRC cells after transfection with BRG1 siRNA2 were determined by Western blotting; the statistical analysis is shown. (B and C) Western blotting and RT‒qPCR were used to detect the expression levels of BRG1 and MMP9 after transfection with LDHA and LDHB siRNAs. (D) P300 and HDAC3 Wprotein levels in LoVo cells after transfection with the BRG1 siRNA2 were determined by Western blotting. (E) Western blotting was used to detect the MMP9 protein levels after ERG knockdown. (F) Western blotting was used to detect the MMP9 protein levels after ERG overexpression. (G) Western blotting was used to detect the P300 protein levels after ERG knockdown. (H) Images showing the enrichment efficiencies of BRG1, P300 and ERG for the lncRNA STEAP3-AS1 obtained by RIP. (I) RT‒qPCR was used to detect the expression levels of the lncRNA STEAP3-AS1 in cells after transfection with the P300 siRNA. (J) IF staining was performed to detect the subcellular localization of ERG proteins and the lncRNA STEAP3-AS1. (K) Western blot analysis of MMP9 protein levels after the overexpression of lncRNA STEAP3-AS1 and the knockdown of BRG1, ERG or P300. (L) ChIP‒qPCR was used to detect the enrichment efficiency of BRG1, ERG, P300 and H3K18la at four sites in the promoter region of MMP9 in LoVo cells.


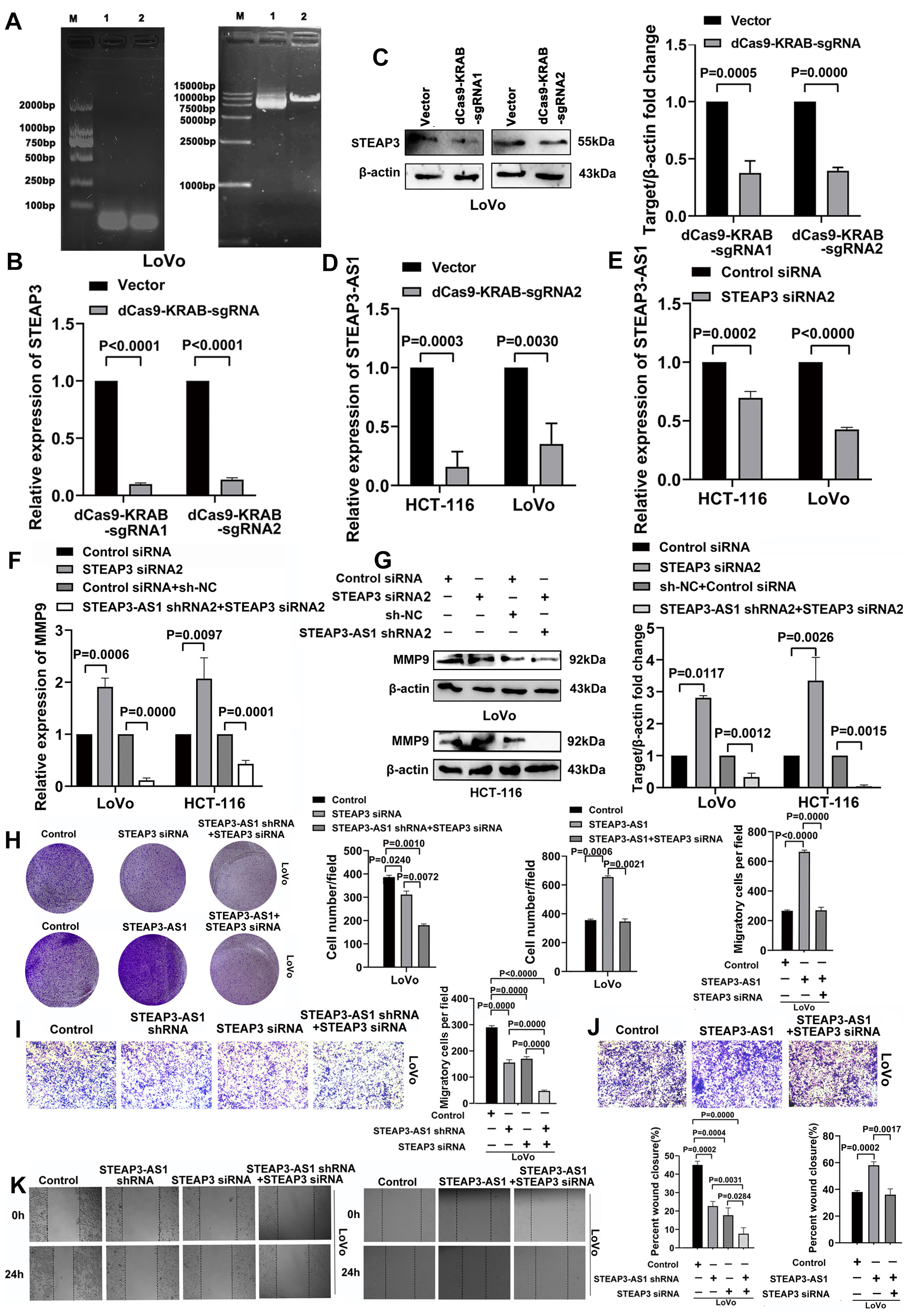


**Figure S6. Related to Figure 6.** The lncRNA STEAP3-AS1 interacts with the parental gene STEAP3 to promote the proliferation and migration of CRC cells. (A) Identification of the DNA fragment size of the sgRNA and restriction enzyme vector by agarose gel electrophoresis. (B and C) RT‒qPCR and Western blotting were used to detect the expression levels of STEAP3 in LoVo cells after transfection with the STEAP3 sgRNA plasmid. (D and E) RT‒qPCR was used to detect the expression levels of the lncRNA STEAP3-AS1 in cells after transfection with the STEAP3 sgRNA plasmid and STEAP3 siRNA. (F and G) RT‒qPCR and Western blotting were used to detect the expression levels of MMP9 after the coknockdown of lncRNA STEAP3-AS1 and STEAP3. (H) The proliferation of LoVo cells was evaluated by colony formation assays. (I and J) Representative images showing the migration of LoVo cells on the membrane and statistical analysis. Scale bars, 200 μm. (K) The migratory ability of LoVo cells was evaluated by wound healing assays. Scale bars, 200 μm.


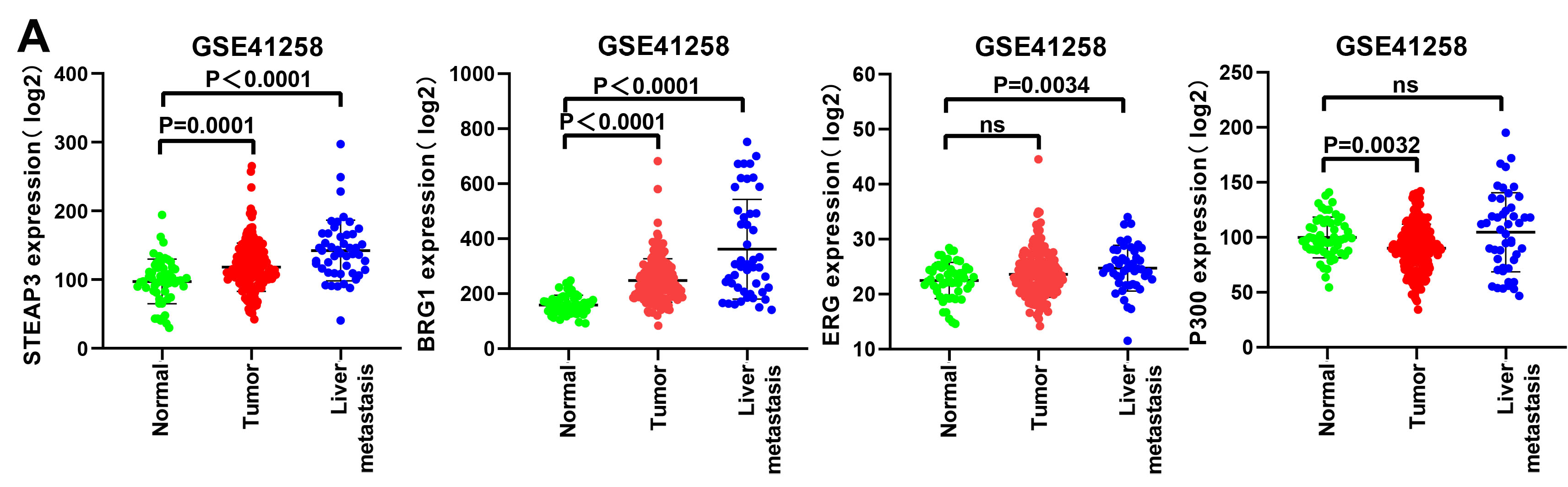


**Figure S7. Related to Figure 7.** Analysis of the expression of STEAP3, lncRNA STEAP3-AS1, BRG1, ERG, and P300 in colorectal cancer tissues. (A) Analysis of the expression levels of STEAP3, BRG1, ERG, and P300 in normal colorectal mucosa, CRC primary tumours and patient-matched liver metastases in the GEO dataset (GSE41258).

**Supplementary Materials and Methods**

**Histology and immunofluorescence staining of CRC organoids**

For H&E and IHC staining, the paraffin blocks were cut into 5-μm sections. For IHC, the sections were blocked with goat serum for 1 h and then incubated overnight at 4 °C with the following antibodies: Ki-67 (1:2000, Proteintech) and BRG1 (1:200, Proteintech). The secondary antibody used was a horseradish peroxidase (HRP)-conjugated goat anti-rabbit antibody. The diaminobenzidine (DAB) chromogen (Thermo Fisher Scientific) was used for detection, and haematoxylin was used as a counterstain. Images were acquired using an Olympus microscope. The expression levels of related proteins in the histological sections were measured in a blinded manner. Organoids were cultured on round coverslips in 24-well plates. Fixation was performed usingan incubation with 4% paraformaldehyde for 20 min, followed by permeabilization with 0.5% Triton X-100 for 30 min. Blocking was performed with goat serum for 30 min, after which the organoids were incubated with the following primary antibodies overnight: β-tubulin (1:50, PTMBIO) and CK20 (1:1000, Proteintech). The secondary antibodies, Alexa Fluor 488-conjugated goat anti-mouse (1:200, Proteintech, RGAM002) and Alexa Fluor 594-conjugated goat anti-rabbit (1:200, Proteintech, RGAR004), were applied for 3 h. DAPI was used as a counterstain for the nuclei. Imaging was performed using an Olympus confocal microscope.

**RNA sequencing**

For quality control, the RNA ScreenTape Kit (Agilent Technologies), RNA ScreenTape Kit (Agilent Technologies), D1000 Screen-Tape Kit (Agilent Technologies), Qubit RNA HS Assay Kit (Invitrogen), and Qubit DNA HS Assay Kit (Invitrogen) were used for specific purposes. For mRNA library preparation, a KAPA Stranded mRNA-seq kit with mRNA capture beads (Kapa Biosystems) was used. Briefly, 1 μg was used for library construction, and the library was eluted in 20 μl of elution buffer. Libraries were adjusted to 10 mM, and 10 μl of each sample was collected and pooled in one tube. A pool of multiplex samples (1.5 pM including 1.5% PhiX) was loaded into the NextSeq 500/550 High Output v2 kit (75 cycles) cartridge (Illumina) and loaded on a NextSeq 500 System (Illumina), with 75 cycles and single-read sequencing conditions. The sequencing data were aligned to the Ensembl *Mus musculus* reference genome, revision 92 (mm10), and the read counts per gene were normalized to the sequencing depth and gene length as fragments per kilobase per million reads mapped (FPKM). Finally, Bowtie software was used to construct the reference genome index, and HISAT2 software was used to align the clean reads to the reference genome. The differential expression analysis of the RNA-seq data was performed using DESeq2. Genes with a [log2 (fold change)] ≥1 and a q value ≤0.05 were considered differentially expressed genes. The Gene Ontology analysis was performed using the DAVID tool.

**ChIP assay**

HCT-116 and LoVo cells were seeded in 10 cm dishes, incubated with 1% formaldehyde at room temperature for 10 min, and then collected. The samples were sonicated and centrifuged at 15,000 × g and 4 °C for 10 min. Supernatants were immunoprecipitated with antibodies specific for rabbit IgG, BRG1, ERG, P300, or H3K18la in the presence of Protein A beads at 4 °C overnight. DNA was purified using a spinning filter and eluted with 50 μL of (dd)H_2_O. The promoter region of MMP9 was amplified using polymerase chain reaction (PCR). The primer sequences are listed in Supplementary Table S2. The PCR conditions were as follows: 40 cycles of amplification at 95 °C for 30 s, 62 °C for 60 s, and 72 °C for 30 s. Agarose gel electrophoresis (2%) was used to analyse the PCR products.

**siRNA transfection and lentiviral transduction**

HCT-116 and LoVo cells were seeded in 6-well plates and transfected with small interfering RNAs (siRNAs) or lentiviral vectors (LVs). siRNAs (80 pmol) were transfected into HCT-116 and LoVo cells using the GP-transfect-Mate system from Gene Pharma (Shanghai, China) according to the manufacturer's protocol. The siRNA sequences were designed and synthesized by Gene Pharma (Shanghai, China). The pCMV-EGFP-puro vector and pCMV-lncRNA STEAP3-AS1-EGFP-puro vector, which encode the full-length human lncRNA STEAP3-AS1, were obtained from Gene Pharma (Shanghai, China). HCT-116 and LoVo cells were infected with the lentivirus at an MOI = 100 in RPMI-1640 and DMEM containing polybrene (5 g/mL). The sequences of the siRNAs are provided in [Supplementary Table S1](https://www.ncbi.nlm.nih.gov/pmc/articles/PMC10007902/#iovs-64-3-16_s002).

**Co-IP assays**

For co-IP assays, HCT-116 and LoVo cells were collected by scraping, followed by two washes with ice-cold PBS. The cell pellet was suspended in 900 μl of ice-cold lysis buffer (20 mM Tris-HCl, pH 8.0, 150 mM NaCl, 1 mM EDTA, 0.5% NP40, 10% glycerol, and 1× protease inhibitor) and rotated at 4 °C for 1 h. The lysate was cleared by centrifugation for 20 min at 4 °C and 20,000 × g. The supernatant was incubated with 2–5 μg of antibody (BRG1 and ERG) for each IP reaction (including IgG as a negative control), followed by rotation overnight at 4 °C. Protein A/G magnetic beads (Smart Lifesciences, blocked with 1 mg/ml BSA for 1 h) were added to the samples, and the mixture was rotated for 3 h at 4 °C. After the incubation, samples containing the beads were collected using a magnetic rack, and the beads were washed four times with lysis buffer. Finally, the samples were collected by adding 100 μl of 1× SDS loading buffer, followed by Western blot analysis.

**RNA Pull-Down Assays**

RNA pull-down assays were performed using a Magnetic RNA‒Protein Pull-Down Kit (Guangzhou, China). The lncRNA STEAP3-AS1 biotin-labelled probe was synthesized by Gene Pharma (Shanghai, China). Total protein was extracted from 2×10^7^ HCT-116 and LoVo cells. RNA probes of corresponding quality were obtained according to the ratio of length to mass, denatured, added to the RNA structure buffer, and incubated at room temperature for 20 min to form the RNA secondary structure. The RNA probe was incubated with washed magnetic beads at 25 °C for 30 min to form a complex. The probe–magnetic bead complex was mixed with the protein sample and incubated with rotation at 4 °C overnight. The magnetic beads were washed, the eluant (protein elution buffer and DTT) was added, and the mixture was incubated at 37 °C for 2 h to elute the precipitated protein. Proteins were subjected to SDS‒PAGE followed by Western blot analysis.

**Quantitative Real-Time PCR**

Total RNA was isolated from cultured cells using TRIzol (Invitrogen). The concentrations of RNA were determined using a NanoDrop 2000 (Thermo Scientific). The RNA was reverse transcribed into cDNA using a PrimeScript RT Reagent Kit (Takara). qPCR primers were synthesized by Sangon Biotech (Shanghai, China), and the reaction was performed with ChamQ Universal SYBR qPCR Master Mix (Vazyme). All the primers used are listed in Supplementary Table S2. The results were normalized to those of GAPDH to analyse relative gene expression using the 2^-△△CT^ method. All quantitative real-time PCR experiments were performed in triplicate.

**Cell Counting Kit-8 and colony formation assays**

Human colon cancer HCT-116 and LoVo cells were plated into 96-well plates (1 × 10^3^ cells/well) for Cell Counting Kit-8 (CCK-8) assays. CCK-8 solution (MedChemExpress, China) was added to each well at 24, 48, and 72 hours. After an incubation for 2 h, the optical density (OD) at 450 nm was measured with a Multiskan FC Microplate Photometer (Thermo Scientific). For the colony formation assays, HCT-116 and LoVo cells were digested and plated into 6-well plates (2 × 10^3^ cells/well). After being cultured for 14 days, the colonies were fixed with methanol for 30 min and then stained with 0.5% crystal violet (5 mg/ml) for another 15 min at RT. Colonies were counted with ImageJ software (NIH Image).

**Wound healing assays**

Colon cancer cells that had been transfected were seeded into 12-well plates, and when the cells were approximately 80% confluent, scratches were made on the confluent monolayer of cells with sterile pipette tips, and the medium was changed to serum-free medium. The scratch areas at 0 h, 12 h and 48 h were recorded under a light microscope, and the scratch area size was analysed using ImageJ software.

**Transwell migration assay**

For the transwell assay, HCT-116 and LoVo cells were digested and plated in a 24-well transwell system, and a polycarbonate filter (8-μm pores, Corning) was used in the transwell assay. A total of 3 × 10^5^ cells suspended in 200 μl of serum-free medium were seeded in the upper compartment, and 800 μl of complete medium supplemented with 10% FBS was added to the lower chamber. After an incubation at 37 °C with 5% CO_2_ for 24 h, the cells that had not migrated were removed from the upper surface of the membrane, while the cells that migrated to the lower surface of the membrane were fixed with 4% paraformaldehyde for 30 min and stained with 0.5% crystal violet for 15 min. The cells that migrated to the lower chamber were imaged and counted.

**Organoid lentiviral and BRG1 siRNA infection**

On the day of infection, the organoids were incubated with 200 μl of concentrated virus in infection medium for 24 h. Infected organoids were collected, embedded in Matrigel and grown in Colon Cancer Organoid Culture Medium for ten days. Bright-field and GFP imaging were performed using a fluorescence microscope. Transient transfection of the BRG1 siRNA was performed using Lipofectamine 2000 (Invitrogen) according to the manufacturer's instructions. The growth of the organoids was continuously observed for 10 days.

**RNA fluorescence in situ hybridization (FISH)**

A FISH in situ hybridization kit with the Gemma gene (GenePharma, China) was used for in situ hybridization. Briefly, the appropriate number of cells was seeded onto a cell crawl within a 24-well plate. The cells were fixed with 4% paraformaldehyde and permeabilized with 0.5% Triton X-100. Paraffin-embedded tissue sections were first rehydrated through a series of solutions, such as xylene. Then, the tissue sections were digested with proteinase K. The cells and sections were sealed with a hybridization solution. The probe mixture for the lncRNA STEAP3-AS1 was then added and incubated at 37 °C for 16 h. Then, the cells were washed with a preprepared mixture, and the nuclei were stained with DAPI. The samples were washed with PBS 3 times between each staining step. The cells were observed using a fluorescence microscope.

**RNA Immunoprecipitation**

A Bersinbio RIP kit (Bersinbio, China) was used. After lysis and DNA removal, the cell lysates were mixed with 5 μg of ERG antibody (Abcam, UK), BRG1 antibody (Proteintech, China), H3K18la antibody (PTM BIO, China), P300 antibody (Abcam, UK) and IgG antibodies and incubated at 4 °C for 16 h. The equilibrated Protein A/G magnetic beads were added and incubated at 4 °C for 1 h. The protein‒RNA complexes pulled down by the magnetic beads were washed with a mixture containing proteinase K and RNA was extracted; the extracted RNA was reverse-transcribed into cDNA. The enrichment efficiency was detected using RT‒qPCR.

**Western blot**

The human colon cancer cell lines HCT-116 and LoVo were lysed in RIPA lysis buffer supplemented with 1 mM [PMSF](https://www.sciencedirect.com/topics/medicine-and-dentistry/pmsf) (Cat. No. BC3710, Solarbio, China) and protease inhibitors on ice. The total protein concentration was quantified using the BCA method in lysis buffer (Cat. No. SW101, Seven, China), and proteins were separated on SDS‒PAGE gels and electroblotted onto a nitrocellulose filter membrane (PALL Gelman Laboratory). The membranes were blocked with 5% skim milk for 1 h at room temperature and then incubated with pan Kla (PTM BIO, PTM-1401, 1:1000), BRG1 (Proteintech, 21634-1-AP, 1:1000), ERG (Abcam, ab133264, 1:1000), P300 (Abcam, ab14984, 1:1000), MMP9 (Proteintech, 10375-2-AP, 1:1000), H3K18la (PTM BIO, PTM-1406RM, 1:1000), β-actin (ZSGB-BIO, TA-09, 1:1000), histone H3 (PTM BIO, PTM-1002RM, 1:1000), STEAP3 (Abcolonal, A0683, 1:800), SMARCA1 (Abcolonal, A10248, 1:1000), and ARID1A (Proteintech, 30304-1-AP, 1:1000) antibodies diluted in 5% skim milk. All the membranes were incubated with the antibodies overnight. The membranes were subsequently washed three times with 1× TTBS for 15 min. Then, each membrane was incubated with goat anti-rabbit IgG (Proteintech, SA00001-2, 1:2000) and goat anti-mouse IgG (Proteintech, SA00001-1, 1:2000) for 1.5 h and washed three times with 1 × TTBS for 15 min. The membranes were then sequentially exposed to an enhanced [chemiluminescence](https://www.sciencedirect.com/topics/biochemistry-genetics-and-molecular-biology/chemoluminescence) system and X-ray film. The intensities of the bands were measured using ImageJ software.

**Statistical analysis**

Each experiment was performed at least three. Data are presented as mean ± SD. Differences between the two groups were assessed using a two-tailed Student’s t-test. Multiple group comparisons were performed using one-way analysis of variance (ANOVA). The paired samples were analyzed using paired t-tests. A p value of less than 0.05 was defined as statistically significant (*P < 0.05, **P < 0.01, ***P < 0.001). All statistical analyses were performed using the GraphPad Prism software (version 8.0).

**Supplementary Tables**

**Supplementary Table S1. shRNA and siRNA sequences used in this study.**

| shRNA or siRNA name | Sequence (5' to 3') |
| --- | --- |
| STEAP3-AS1 shRNA1 | 5′-GGGAACAAGCTGAACACAACA-3′ |
| STEAP3-AS1 shRNA2 | 5′-GCACCTTTAAACTGTCCTACA-3′ |
| control shRNA | 5′-TTCTCCGAACGTGTCACGT-3′ |
| siSTEAP3-1 | 5´-GAGUUCAGCUUCGUUCAGUTT-3´ |
| siSTEAP3-1 | 5´-GCUUCUAUGCCUACAACUUCG-3´ |
| siBRG1-1 | 5´-CAGAUCAUGGCCUACAAGAUG-3´ |
| siBRG1-2 | 5´-GCAUUCUCCAGCAUGCCAATT-3´ |
| siERG-1 | 5´-GACCAAAAGCAAGACAAAUGA-3´ |
| siERG-2 | 5´-GACCAGUCGUUGUUUGAGUTT-3´ |
| siLDHA | 5´-UGUAGCAGAUUUGGCAGAGAGUAUA-3´ |
| siLDHB | 5´-GAGCUUAUUUCUUCAGACACCUAAA-3´ |
| control siRNA | 5´-UUCUCCGAACGUGUCACGUTT-3´ |

**Supplementary Table S2.** Primers sequences used in this study.

| Gene name | | Sequence (5' to 3') | | Amplified  size （bp） | |
| --- | --- | --- | --- | --- | --- |
| STEAP3-AS1 | | F: 5’-TGCTGGGAAAGGGAACTCTG-3’ | | 149 | |
|  |  | R: 5’-TCCTGGTCATCAAACACCCAG-3’ | |  |  |
| BRG1 | | F: 5’-CCTGGCATCAGTAGCATCTGTAA-3’ | | 79 | |
|  |  | R: 5’-TCGTGTGTTCCCCAATTCG-3’ | |  |  |
| CEACAM22P | | F:5’-CTCGTGGTTGGCGTGGCTTATG-3’ | | 120 | |
|  | | R:5’-CTGGGCAAGGAGGTGGGAGTAG-3’ | |  | |
| C22orf34 | | F:5’-ATTCTTGGAGCAGCATCGGCATC-3’ | | 132 | |
|  | | R:5’-GAGTTGGCAGCAGGTTCCTTGG-3’ | |  | |
| SPARC | | F:5’-GGTGTGCGAGCTGGATGAGAAC-3’ | | 142 | |
|  | | R:5’-TGTGGCAAAGAAGTGGCAGGAAG-3’ | |  | |
| NAALADL1 | | F:5’-CCCTCCCTTTACTCCAACCCTCTC-3’ | | 86 | |
|  | | R:5’-TCCTGTGGTAGTGCCCTCTTCTG-3’ | |  | |
| ERG | | F:5’-GGAGTGGGCGGTGAAAGAATATGG-3’ | | 139 | |
|  | | F:5’-GGAGTGGGCGGTGAAAGAATATGG-3’ | |  | |
| MARK2P9 | | F:5’-AGGAATAAATTCGCCTTTCGCAACAAG-3’ | | 123 | |
|  | | R:5’-GGATAACTCGCAGACGCCATACATC-3’ | |  | |
| SLAIN1 | | F:5’-TGCAGCTTCTGGGATAATGG-3’ | | 144 | |
|  | | R:5’-AGAGTGCTGAGTGAAGACAGAGG-3’ | |  | |
| MMP9 | | F:5’-CCCCTTCACTTTCCTGGGTAA-3’ | | 151 | |
|  | | R:5’-CGCCACGAGGAACAAACTGT-3’ | |  | |
| ERG | | F:5’-GGAGTGGGCGGTGAAAGAATATGG-3’ | | 139 | |
|  | | F:5’-GGAGTGGGCGGTGAAAGAATATGG-3’ | |  | |
| FLI1 | | F:5’-GGATGGCAAGGAACTGTGTAA-3’ | | 127 | |
|  | | R:5’-GGTTGTATAGGCCAGCAG-3’ | |  | |
| SPI1 | | F:5’-GCCAAACGCACGAGTATT-3’ | | 180 | |
|  | | R:5’- GCTCCATGTGGCGGTAGA-3’ | |  | |
| ELF1 | | F:5’-GTTCCACAATTACGGCAG-3’ | | 101 | |
|  | | R:5’-TGTCCAACAGAACGACCT-3’ | |  | |
| ETS1 | | F:5’-ACGATAGTTGTGATCGCCTCA-3’ | | 242 | |
|  | | R:5’-CCATAGCTGGATTGGTCCACT-3’ | |  | |
| GAPDH | | F:5’-CCTCAAGATCATCAGCAAT-3’ | | 141 | |
|  | | R:5’-CCATCCACAGTCTTCTGGGT-3’ | |  | |
| ChIP | |  | |  | |
| MMP9 P1  (-355 to -152)  MMP9 P2  (-1234 to -1055)  MMP9 P3  (-2200 to -2015)  MMP9 P4  (-2482 to -2253) | | F: 5’-ctggaggctttcagaccaag-3’ | |  | |
|  |  | R: 5’-aagggcttacaccacctcct-3’  F: 5’-cagggctggagaactgaaag-3’  R: 5’-cctgccaaaagaccatgatt-3’  F: 5’-acctgggtcatcacagttcc-3’  R: 5’-agcctctcgtttcatcctca-3’  F: 5’-ttcctaaaagcccgtgtctg-3’  R: 5’-acaggggcagcacataaaac-3’ | |  |  |
|  | BRG1 primer of R-loop | | Sequence (5' to 3') | |  |
|  | TSS | | F:5’ -GGGGTGGATTGTAAGGGATA-3’ | |  |
|  |  | | R:5’- ACTTTGTAGGGGAGATGAGGAC-3’ | |  |
|  | TES | | F:5’- ATGATGAAACCCTGTCTCTACTGAA-3’ | |  |
|  |  | | R:5’ -GCAGTGGCACAATCTCCTCTC-3’ | |  |

**The FISH probe sequence is as follows:**

| STEAP3-AS1 | 5′-FAM-AACTGCAGATTCTGTTCCAATGGACTACAGCACCTTAAATGCAGGAGAGAGACTTGGCTTTTAGAAGTAGGTTCTGGGGGAATAATACAAGAACTGCTCCAAGATGCAAA-3′ |
| --- | --- |
