# Supplementary material for: The LncRNA STEAP3-AS1 promotes liver metastasis in colorectal cancer by regulating histone lactylation through chromatin remodelling
Source: J Exp Clin Cancer Res. 2025 Jul 15;44:205. doi: 10.1186/s13046-025-03461-0 (PMC12261760; doi:10.1186/s13046-025-03461-0)

Figure 2

Figure 2 E

HCT-116-MMP9

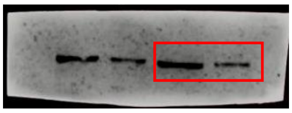

HCT-116-β-actin

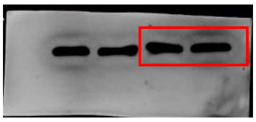

LoVo-MMP9

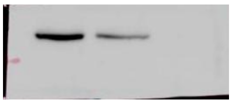

LoVo-MMP9

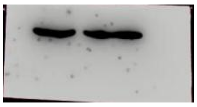

Figure 2 H

HCT-116-BRG1

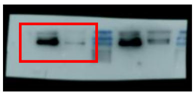

HCT-116-P300

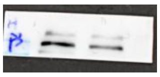

HCT-116-ARID1a

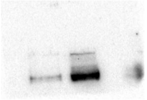

HCT-116-SMARCD1

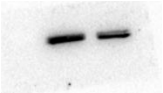

HCT-116-actin

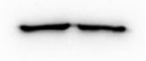

LoVo-BRG1

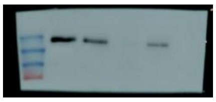

LoVo-p300

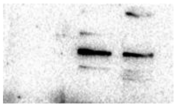

LoVo-ARID1a

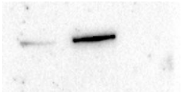

LoVo-SMARCD1

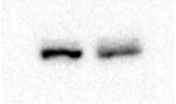

LoVo-actin

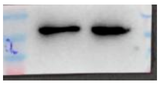

Figure 2 I

HCT-BRG1

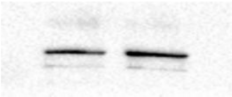

HCT-MMP9

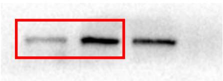

HCT-ERG

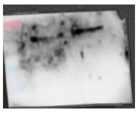

HCT-actin

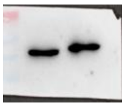

LoVo-BRG1

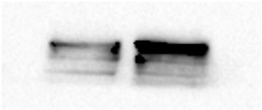

LoVo-MMP9

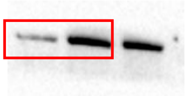

LoVo-ERG

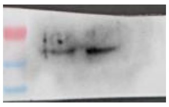

LoVo-actin

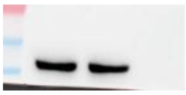

Figure 2 K

PDO-BRG1

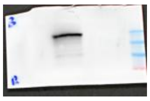

PDO-actin

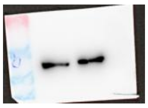

Figure 3

Figure 3 C

HCT-116-STEAP3

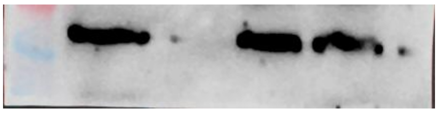

LoVo-STEAP3

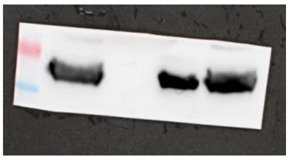

Figure 3 I

HCT-116-BRG1

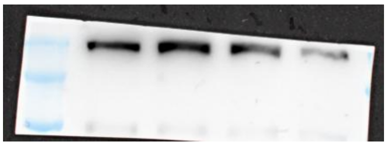

HCT-116-β-actin

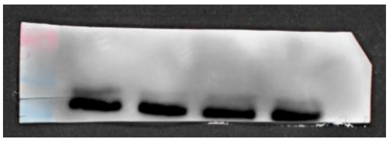

LoVo-BRG1

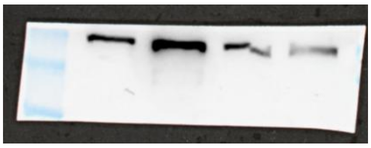

LoVo-β-actin

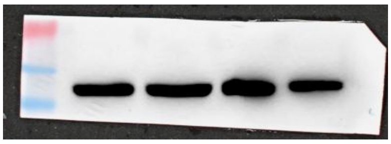

Figure 3 J

HCT-116-BRG1

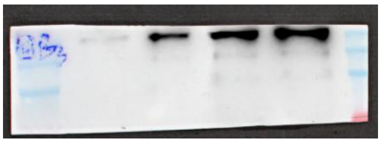

LoVo-β-actin

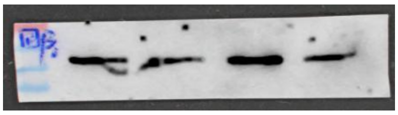

LoVo-BRG1

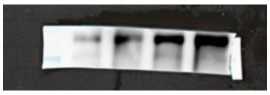

LoVo-β-actin

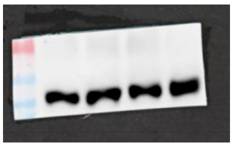

Figure 4

Figure 4 A

succinylation

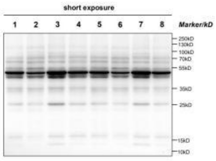

β-hydroxybutyryllysineation

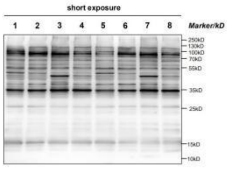

crotonylation

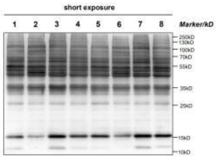

Lactylation

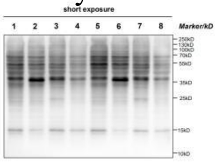

acetylation

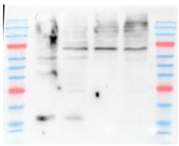

Group: 1.LoVo(Control shRNA1), 2.Lovo(STEAP3-AS1 shRNA1), 3.HCT-116(Control shRNA), 4.HCT-116(STEAP3-AS1 shRNA1), 5.LoVo(Control shRNA), 6.Lovo(STEAP3-AS1 shRNA1), 7.HCT-116(Control shRNA), 8.HCT-116(STEAP3-AS1 shRNA).

Figure 4 C

HCT-116-actin

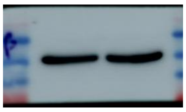

HCT-116-H4K5la

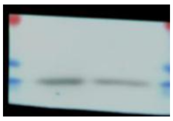

HCT-116-H3K9la)

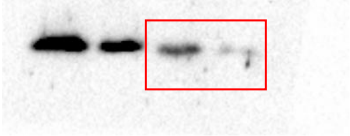

HCT-116-H3K18la

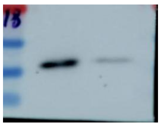

LoVo-actin

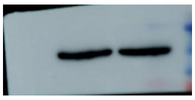

LoVo-H4K5la

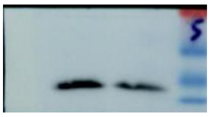

LoVo-H3K9la

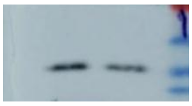

LoVo-H3K18la

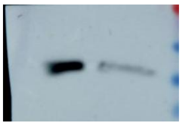

Figure 4 G

HCT-116-H3K18la+HG

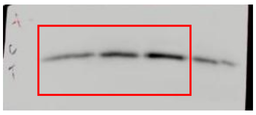

HCT-116-H3K18la+Rotenone

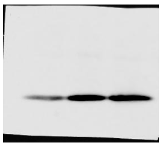

HCT-116-H3K18la+lactate

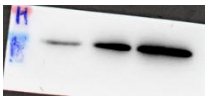

HCT-116-H3+HG

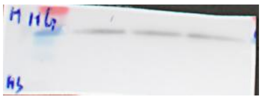

HCT-116-H3+Rotenone

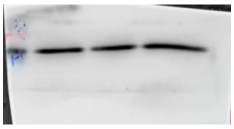

HCT-116-H3+lactate

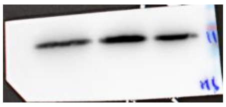

Lovo-H3K18la+HG

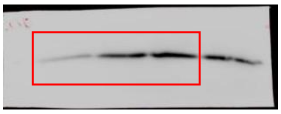

Lovo-H3K18la+Rotenone

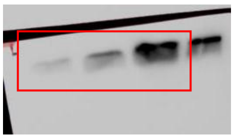

Lovo-H3K18la+lactate

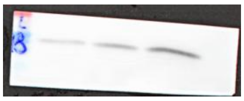

LoVo-H3+HG

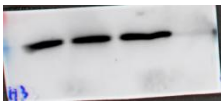

Lovo-H3+Rotenone

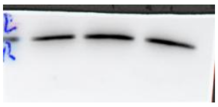

Lovo-H3+lactate

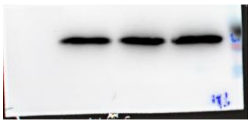

Figure 4 H

H3K18la

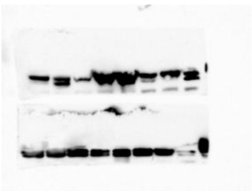

actin

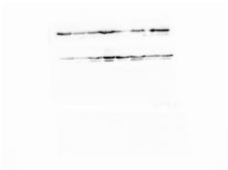

Histone-H3

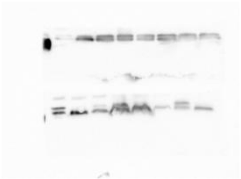

Figure 5

Figure 5A

HCT-116-ERG

HCT-116-MMP9

HCT-116-BRG1

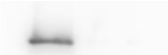

HCT-116-H3K18la

HCT-116-β-actin

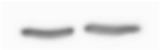

HCT-116-H3

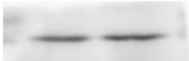

LoVo-ERG

LoVo-BRG1

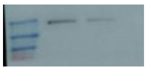

LoVo-MMP9

LoVo-H3K18la

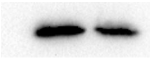

LoVo-β-actin

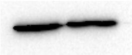

LoVo-H3

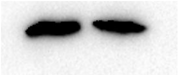

Figure 5 B

HCT-116-MMP9+HG

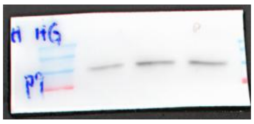

HCT-116-BRG1+HG

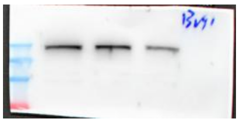

HCT-116-β-actin+HG

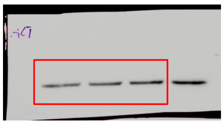

HCT-116-MMP9+Rotenone

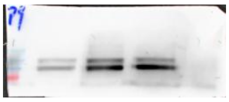

HCT-116-BRG1+Rotenone

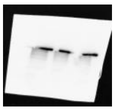

HCT-116-β-actin+Rotenone

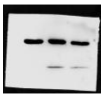

HCT-116-MMP9+lactate

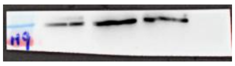

HCT-116-BRG1+lactate

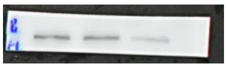

HCT-116-β-actin+lactate

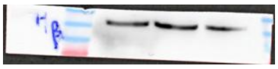

LoVo-MMP9+HG

LoVo-BRG1+HG

LoVo-β-actin+HG

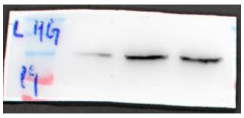
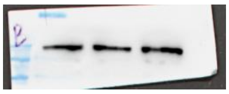
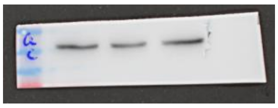

LoVo-MMP9+Rotenone   LoVo-BRG1+Rotenone   LoVo-β-actin+Rotenone

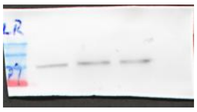
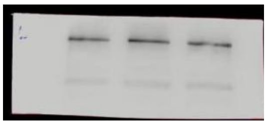
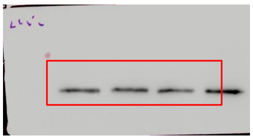

LoVo-MMP9+lactate   LoVo-BRG1+lactate   LoVo-β-actin+lactate

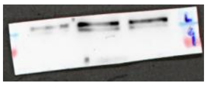
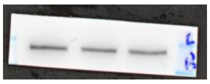
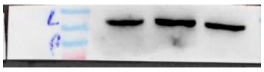

Figure 5 C

HCT-116-P300   HCT-116-HDAC3   HCT-116-β-actin

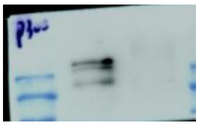
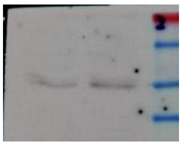
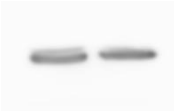

LoVo-P300   LoVo-HDAC3   LoVo-β-actin

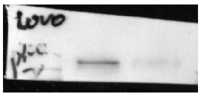

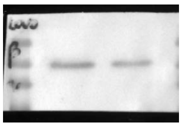

Figure 5 D

HCT-116-MMP9   HCT-116-ERG   HCT-116-β-actin

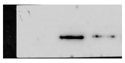
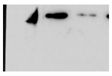
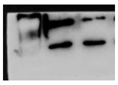

LoVo-MMP9   LoVo-ERG   LoVo-β-actin

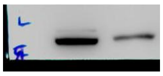
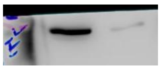
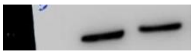

Figure 5 E

HCT-116-P300   HCT-116-β-actin

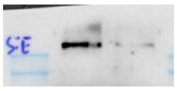
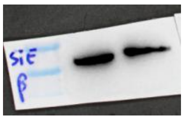

LoVo-P300   LoVo-β-actin

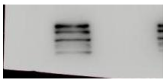 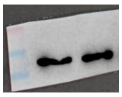

Figure 5 G

IP: BRG1

HCT-116-BRG1 HCT-116-P300 HCT-116-HDAC3

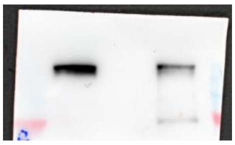 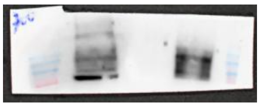 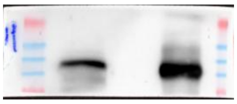

IP:P300

HCT-116-ERG HCT-116-P300

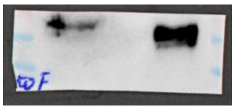 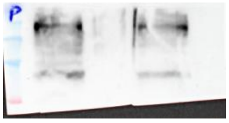

IP:BRG1

LoVo-BRG1 LoVo-P300 LoVo-HDAC3

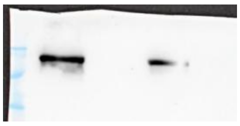 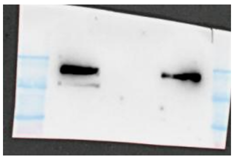 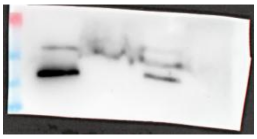

IP:P300

LoVo-ERG LoVo-P300

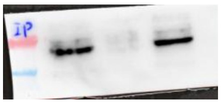 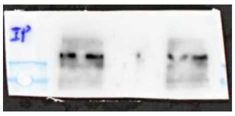

Figure 5 H

HCT-116-P300 HCT-116-β-actin HCT-116-H3K18la HCT-116-H3

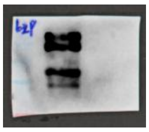 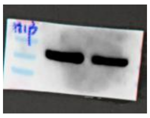 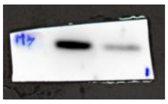 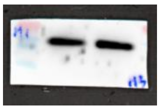

LoVo-P300 LoVo-β-actin LoVo-H3K18la LoVo-H3

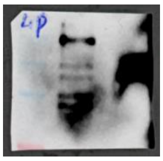 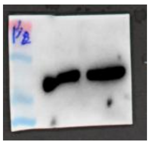 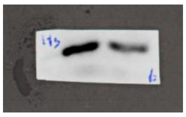 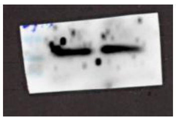

Figure 5 J

HCT-116-MMP9

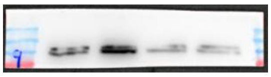

HCT-116-β-actin

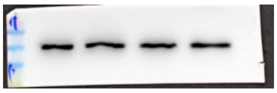

HCT-116-MMP9

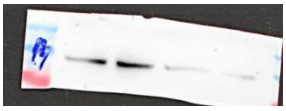

HCT-116-β-actin

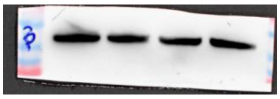

HCT-116-MMP9

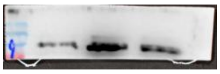

HCT-116-β-actin

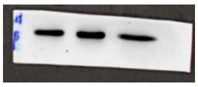

Figure 6

Figure 6C

HCT-116-STEAP3(sgRNA1)

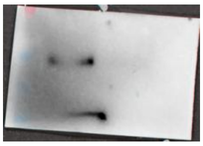

HCT-116-actin(sgRNA1)

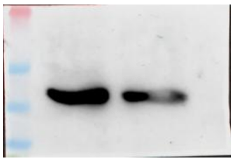

HCT-116-STEAP3(sgRNA2)

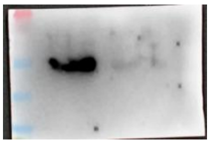

HCT-116-actin(sgRNA2)

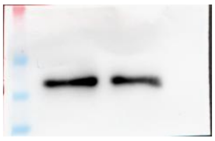

Figure 6H

HCT-116-MMP9

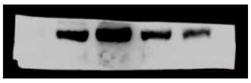

HCT-116-actin

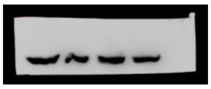

LoVo-MMP9

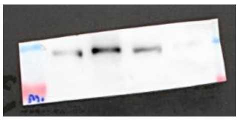

LoVo-actin

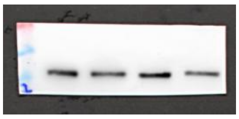

Figure 6J

HCT-116-MMP9

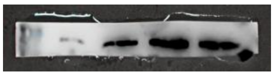

HCT-116-actin

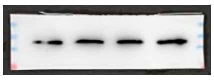

LoVo-MMP9

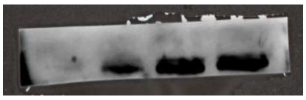

LoVo-actin

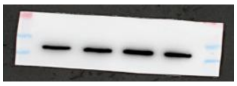

Figure S2. Related to Figure 2

Figure S2G

HCT-116-ERG(sh1) HCT-116-β-actin(sh1) HCT-116-ERG(sh2) HCT-116-β-actin(sh2)

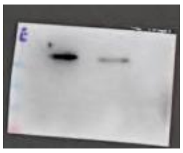
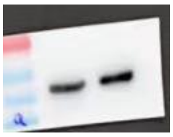
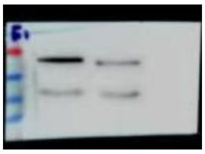
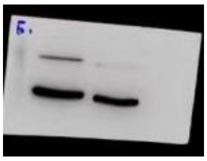

LoVo-ERG(sh1) LoVo-β-actin(sh1) LoVo-ERG(sh2) LoVo-β-actin(sh2)

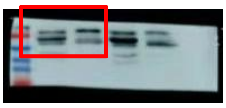
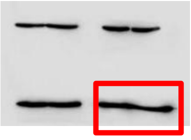
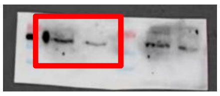
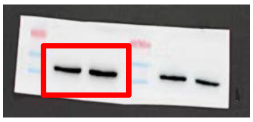

Figure S2H

HCT-116-ERG HCT-116-β-actin LoVo-ERG LoVo-β-actin

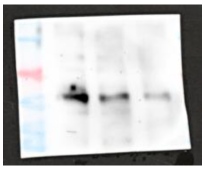
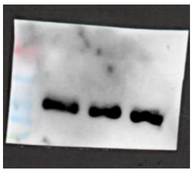
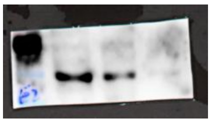
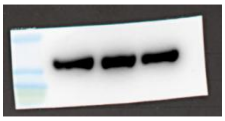

HCT-116-ERG HCT-116-β-actin LoVo-ERG LoVo-β-actin

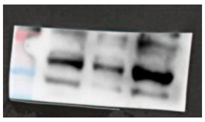
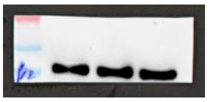
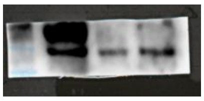
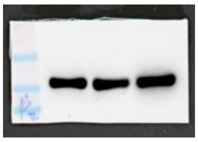

Figure S2I

Patient 1-ERG Patient 2-ERG Patient 3-ERG Patient 4-ERG

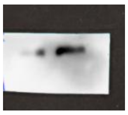
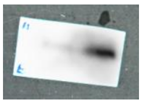
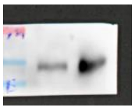
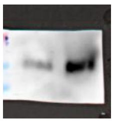

Patient 1-actin Patient 2-actin Patient 3-actin Patient 4-actin

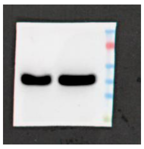
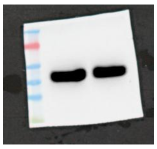
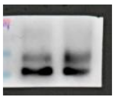
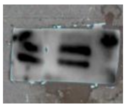

Patient 5-8-ERG

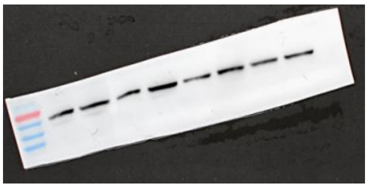

Patient 1-4-actin

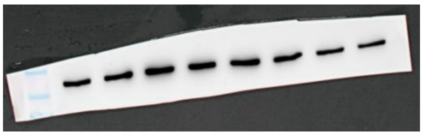

Figure S3. Related to Figure 3

Figure S3F

HCT-116-STEAP3(siRNA1)

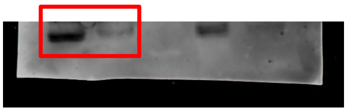

HCT-116-actin(siRNA1)

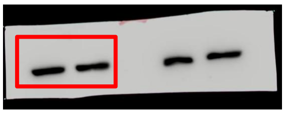

LoVo-STEAP3(siRNA1)

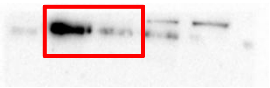

LoVo-actin(siRNA1)

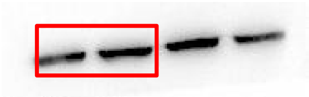

Figure S3G

HCT-116-STEAP3(siRNA2)

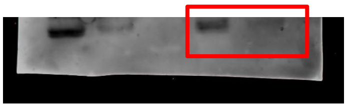

HCT-116-actin(siRNA2)

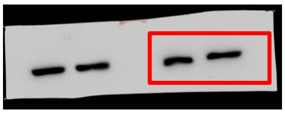

LoVo-STEAP3(siRNA2)

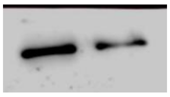

LoVo-actin(siRNA2)

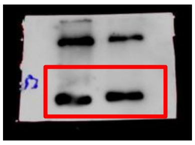

Figure S3H

HCT-116-BRG1

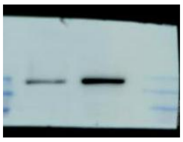

HCT-116-β-actin

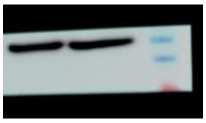

LoVo-BRG1

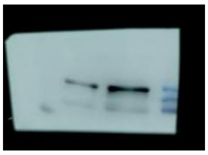

LoVo-β-actin

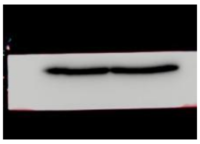

Figure S3I

HCT-116-BRG1

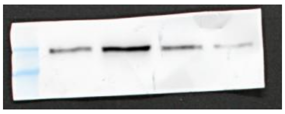

HCT-116-β-actin

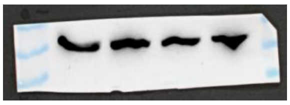

LoVo-BRG1

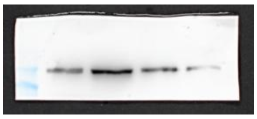

LoVo-β-actin

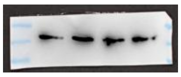

Figure S4. Related to Figure 4

Figure S4A

Pankla

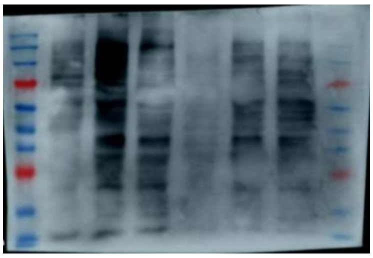

Group： HcoEpic， LoVo， HCT-116， HCT-15， SW480， LS174T

Figure S4 C

HCT-116-β-actin

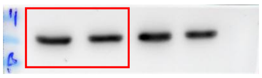

HCT-116-H4K5la

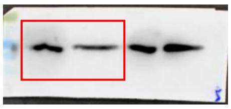

HCT-116-H3K9la

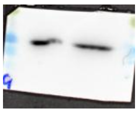

HCT-116-H3K18la

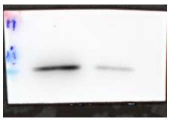

LoVo-β-actin

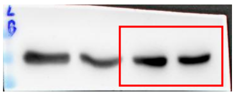

LoVo-H4K5la

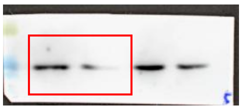

LoVo-H3K9la

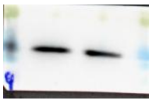

LoVo-H3K18la

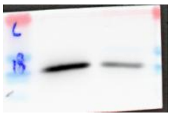

Figure S4 I

H3K18la(#4-7)

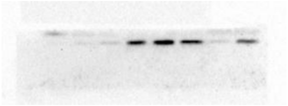

Histone H3(#4-7)

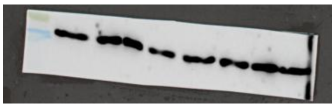

H3K18la(#8-11)

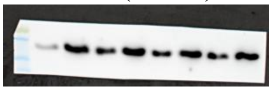

Histone H3(#8-11)

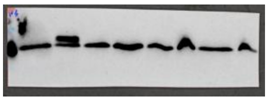

H3K18la(#8-11)

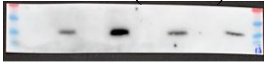

Histone H3(#8-11)

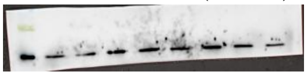

H3K18la(#8-11)

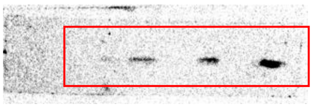

Histone H3(#8-11)

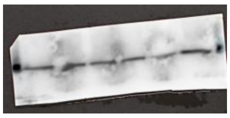

Figure S5. Related to Figure 5

Figure S5A

HCT-116-BRG1

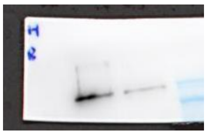

HCT-116-ERG

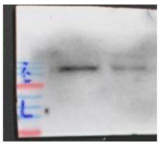

HCT-116-MMP9

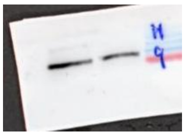

HCT-116-β-actin

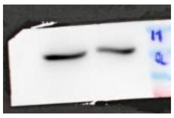

HCT-116-H3K18la

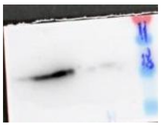

HCT-116-H3

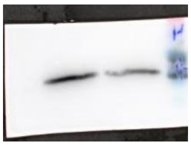

LoVo-BRG1

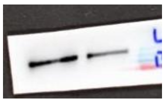

LoVo-ERG

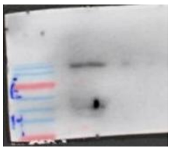

LoVo-MMP9

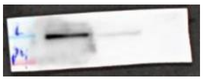

LoVo-β-actin

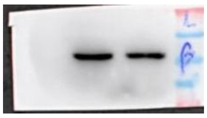

LoVo-H3K18la

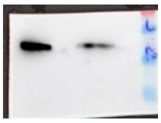

LoVo-H3

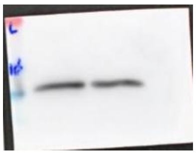

Figure S5 B

HCT-116-BRG1

HCT-116-H3K18la

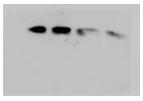

HCT-116-MMP9

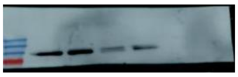

HCT-116-β-actin

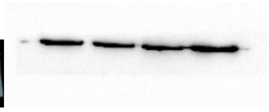

LoVo-BRG1

LoVo-H3K18la

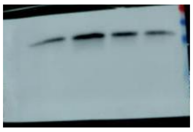

LoVo-MMP9

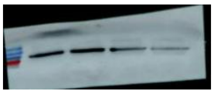

LoVo-β-actin

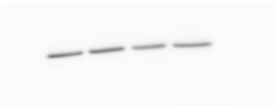

Figure S5 D

HCT-116-P300

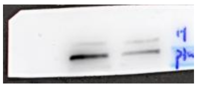

HCT-116-HDAC3

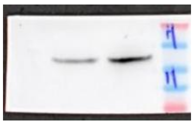

HCT-β-actin

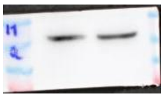

LoVo-P300

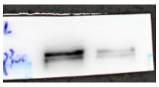

LoVo-HDAC3

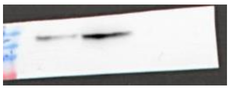

LoVo-β-actin

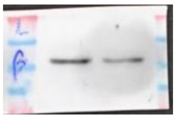

Figure S5 E

HCT-116-ERG

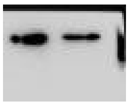

HCT-116-MMP9

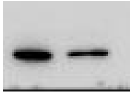

HCT-116-β-actin

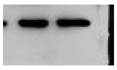

LoVo-ERG

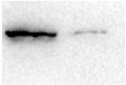

LoVo-MMP9

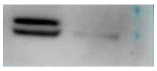

LoVo-β-actin

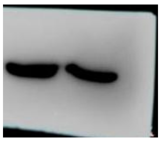

Figure S5 F

HCT-116-P300

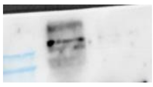

HCT-116-β-actin

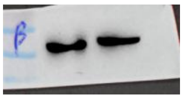

LoVo-P300

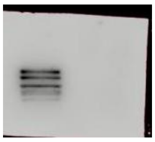

LoVo-β-actin

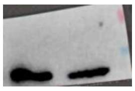

Figure S5 G

RIP-HCT-116

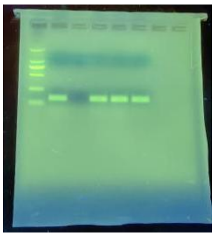

RIP-LoVo

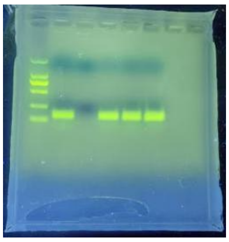

Figure S5 I

HCT-116-ERG

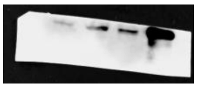

HCT-116-MMP9

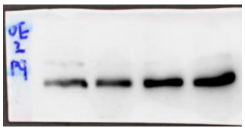

HCT-116-β-actin

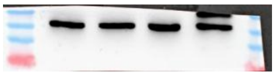

LoVo-MMP9(below)

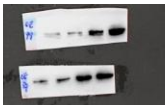

LoVo-ERG

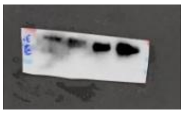

LoVo-β-actin

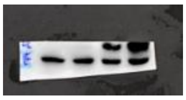

Figure S5 J

LoVo-MMP9

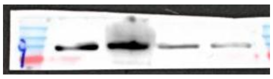

LoVo-β-actin

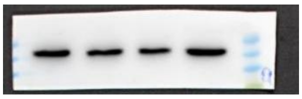

LoVo-MMP9

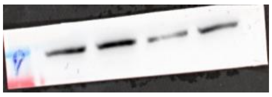

LoVo-β-actin

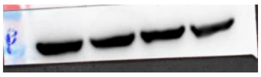

LoVo-MMP9

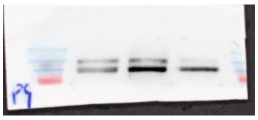

LoVo-β-actin

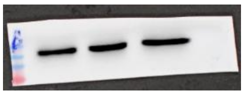

Figure S6. Related to Figure 6

Figure S6A

LoVo-DNA fragments of STEAP3

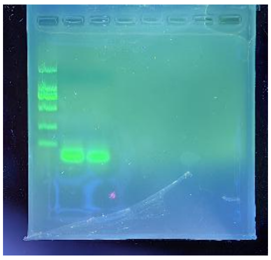

LoVo-enzyme digestion vectors

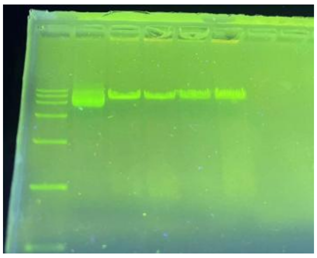

Figure S6C

LoVo-STEAP3(sgRNA1)

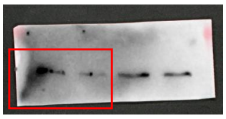

LoVo-actin(sgRNA1)

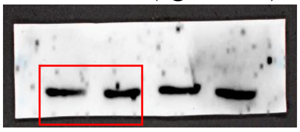

LoVo-STEAP3(sgRNA2)

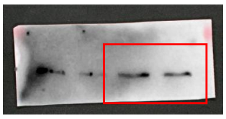

LoVo-actin(sgRNA2)

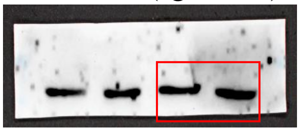

Figure S6G

LoVo-MMP9

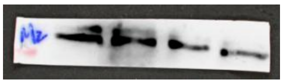

LoVo-MMP9

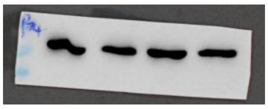

HCT-116-MMP9

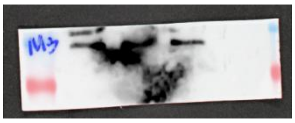

HCT-116-MMP9

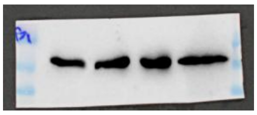

Supplement: Supplementary file 3 — Supplementary Material 3 [file 13046_2025_3461_MOESM3_ESM.pdf]
